# Class I histone deacetylases (HDAC) critically contribute to Ewing sarcoma pathogenesis

**DOI:** 10.1186/s13046-021-02125-z

**Published:** 2021-10-15

**Authors:** Oxana Schmidt, Nadja Nehls, Carolin Prexler, Kristina von Heyking, Tanja Groll, Katharina Pardon, Heathcliff D. Garcia, Tim Hensel, Dennis Gürgen, Anton G. Henssen, Angelika Eggert, Katja Steiger, Stefan Burdach, Günther H. S. Richter

**Affiliations:** 1 Children’s Cancer Research Center and Department of Pediatrics, Klinikum rechts der Isar, Technische Universität München, München, Germany; 2 German Cancer Research Center (DKFZ), Partner Site Munich, München, Germany; 3 Institute of Pathology, School of Medicine, Technische Universität München and Comparative Experimental Pathology (CEP), Technische Universität München, München, Germany; 4 Department of Pediatrics, Division of Oncology and Hematology, Charité - Universitätsmedizin Berlin, Augustenburger Platz 1, Berlin, Germany; 5 Experimental Pharmacology & Oncology Berlin-Buch GmbH, Berlin, Germany

**Keywords:** Ewing sarcoma, Class I HDACs, Expression profiles, Pathogenesis, Targeted therapy

## Abstract

**Background:**

Histone acetylation and deacetylation seem processes involved in the pathogenesis of Ewing sarcoma (EwS). Here histone deacetylases (HDAC) class I were investigated.

**Methods:**

Their role was determined using different inhibitors including TSA, Romidepsin, Entinostat and PCI-34051 as well as CRISPR/Cas9 class I HDAC knockouts and HDAC RNAi. To analyze resulting changes microarray analysis, qRT-PCR, western blotting, Co-IP, proliferation, apoptosis, differentiation, invasion assays and xenograft-mouse models were used.

**Results:**

Class I HDACs are constitutively expressed in EwS. Patients with high levels of individual class I HDAC expression show decreased overall survival. CRISPR/Cas9 class I HDAC knockout of individual HDACs such as HDAC1 and HDAC2 inhibited invasiveness, and blocked local tumor growth in xenograft mice. Microarray analysis demonstrated that treatment with individual HDAC inhibitors (HDACi) blocked an EWS-FLI1 specific expression profile, while Entinostat in addition suppressed metastasis relevant genes. EwS cells demonstrated increased susceptibility to treatment with chemotherapeutics including Doxorubicin in the presence of HDACi. Furthermore, HDACi treatment mimicked RNAi of EZH2 in EwS. Treated cells showed diminished growth capacity, but an increased endothelial as well as neuronal differentiation ability. HDACi synergizes with EED inhibitor (EEDi) in vitro and together inhibited tumor growth in xenograft mice. Co-IP experiments identified HDAC class I family members as part of a regulatory complex together with PRC2.

**Conclusions:**

Class I HDAC proteins seem to be important mediators of the pathognomonic EWS-ETS-mediated transcription program in EwS and in combination therapy, co-treatment with HDACi is an interesting new treatment opportunity for this malignant disease.

**Supplementary Information:**

The online version contains supplementary material available at 10.1186/s13046-021-02125-z.

## Background

Ewing sarcoma (EwS) is a highly malignant bone and soft tissue neoplasia in children and young adults with early metastasis to lung and bone [1]. EwS is defined by specific balanced chromosomal *EWSR1/ETS* translocations giving rise to oncogenic chimeric proteins, the most common being EWS-FLI1 as a consequence of the t(11;22)(q24;q12) translocation [2–4]. Other contributing somatic mutations involved in disease development were only observed at low frequency [5–9].

EWS-FLI1 expression leads to widespread epigenetic changes in promoters, enhancers, and super-enhancers. Such global alterations of histone H3K27-acetylation as well as H3K27-trimethylation associated with an altered HDAC activity seem a general feature of EwS [10, 11]. More than 10 years ago, we identified histone methyltransferase EZH2, part of the polycomb repressor complex (PRC2), to be overexpressed in EwS. RNA interference of EZH2 revealed an EZH2-maintained, undifferentiated reversible, highly malignant, stemness phenotype in EwS [12, 13]. Interestingly, the effects of gene silencing after RNA interference of EZH2 were mimicked by treatment of EwS with different HDAC inhibitors including Trichostatin A (TSA) or Entinostat (MS-275) [12].

There are a number of major classes of HDAC inhibitors including hydroxamic acid-based, cyclic tetra/depsipeptides, amino-benzamide-based, and short-chain fatty acid-derived inhibitors [14]. Entinostat (MS-275), an HDAC1 and HDAC3 inhibitor, was the first amino-benzamide-based HDAC inhibitor to enter clinical trials [15]. Subsequent data revealed a reasonable safety profile and promising efficacy in patients with leukemia, lymphoma, melanoma, prostate cancer, renal cancer, non-small cell lung cancer, and breast cancer, either alone or in combination with other therapies [16–19].

Romidepsin (FK228), a natural product isolated from *Chromobacterium violaceum*, gained approval to treat both cutaneous T-cell lymphoma (CTCL) and peripheral T-cell lymphomas (PTCL) [14]. Modifications to the hydroxamic acid scaffold of this HDAC1 and HDAC2 inhibitor resulted in the discovery and synthesis of PCI-34051 a HDAC8 specific inhibitor, which induced apoptosis in T-cell lymphoma without increasing histone and a-tubulin acetylation [20].

All these more selective HDAC inhibitors mentioned, target a specific group of HDACs belonging to the class I HDAC family comprising HDAC1, 2, 3 and 8. They are ubiquitously expressed, localized predominantly to the nucleus and exhibit selective enzymatic activity toward histone substrates. HDAC1 and HDAC2 are almost identical and often together in repressive complexes such as the sin3, NuRD, CoResT and PRC2 complexes [21]. HDAC3 is expressed in distinct complexes such as the N-CoR–smRT complex, but no complex has been described for HDAC8 so far [22].

In EwS antitumor activity of Entinostat seems mediated via DNA synthesis inhibition, cell cycle arrest, increases in the expression of p21, TGF-βRII, and c-myc, as well as the induction of apoptosis [23, 24]. Recently, it was shown that HDAC3 is a transcriptional target of EWS-FLI1 and that Entinostat inhibits growth via suppressing a previously unexplored EWS-FLI1/HDAC3/HSP90 signaling axis [25]. Welch et al. selected clinically utilized chemotherapy agents and active metabolites including HDAC inhibitor Romidepsin and the reversible LSD1 inhibitor SP2509, both part of the nucleosome remodeling and deactylase (NuRD) co-repressor complex, for tests in EwS cell lines. In two drug experiments, synergy was observed with several combinations, including when SP2509 was paired with topoisomerase inhibitors or Romidepsin [26].

In order to better understand the role of class I HDACs in the pathogenesis of the disease and to develop appropriate approaches for new therapies in EwS, different inhibitors such as TSA, Romidepsin, Entinostat and PCI-34051 were applied, CRISPR/Cas9 class I HDAC knockouts and cells with inducible HDAC specific shRNAs were generated and their influence on gene expression and biological behavior was investigated.

## Material and methods

### Cell lines

EwS cell line A673 was obtained from ATCC (LGC Standards, Teddington, UK). SK-N-MC was purchased from the German collection of Microorganisms and Cell GmbH (Leibniz Institute DSMZ, Braunschweig, Germany). EW7 cell line was kindly provided by O. Delattre (Institut Curie, Paris, France). CHLA-10 were derived from the COG Cell Line & Xenograft Repository (www.cogcell.org). For lentiviral packaging production, HEK 293 T cells were obtained from Dharmacon™ (Colorado, USA). EwS cell lines were cultured in RPMI1640, including 10% heat-inactivated fetal bovine serum and antibiotics (all Life Technologies, Darmstadt, Germany). CHLA-10 cell line required IMDM, 20% heat-inactivated fetal bovine serum, antibiotics and 1% insulin (Life Technologies). Cells were maintained at 37 °C and 5% CO_2_ in a humidified atmosphere. EwS cell lines were routinely checked for purity (e.g. EWS-FLI1 translocation product, surface antigen or HLA-phenotype) and Mycoplasma contamination (MycoAlertTM Mycoplasma Detection Kit, Lonza, Basel, Switzerland) as well as for identity by DNA profiling using 8 different and highly polymorphic short tandem repeat (STR) loci (DSMZ or Eurofins, Ebersberg, Germany).

### Chemical compounds

HDAC inhibitors, TSA, Entinostat (MS-275), Romidepsin (FK228) and PCI-340510 as well as chemotherapeutics Doxorubicin and Vincristine were from Selleckchem (Houston, TX 77014 USA). A-395 was from Sigma-Aldrich (Munich, Germany). The compounds were dissolved in dimethylsulfoxide (DMSO) or DEPC-treated H_2_O, depending on the manufacturer’s instructions, and stored at − 20 °C.

### CRISPR/Cas9 knockouts

CRISPR/Cas9 knockouts of HDAC1 and HDAC2 in EwS cell lines (SK-N-MC, EW7, CHLA-10) were generated using the lentiCas9-Blast vector (plasmid #52962; Addgene, Watertown, MA, USA) and lentiGuide-Puro (Addgene; plasmid #52963). The sgRNA sequence recommendations were used and synthesized from libraries for optimized sgRNA: Brunello and Brie, designed to maximize Rule Set 2 scores and minimize off-target sites with high CFD scores [27]. For lentiviral packaging the Trans-Lentiviral Packaging Kit (ThermoFisher Scientific, Braunschweig, Germany) was used according to the manufacturer’s instructions.

### Lentivirus mediated inducible RNA interference

Doxycycline inducible gene knock downs of either HDAC3, HDAC8 or EWSR1-FLI1 was achieved by the use of pZIP-TRE3G-ZsGreen or cloning into pTRIPZmir30 vector (see Supplementary Information, Additional file 1), respectively. pZIP-TRE3G-ZsGreen were used according to manufacturer’s instructions (shERWOOD UltramiR lentiviral inducible shRNA, bacterial glycerol stock) and obtained from BioCat GmbH (Heidelberg, Germany). For lentiviral packaging the 3rd Generation Packaging Mix & LentiFectin™ Combo Pack Kit (Applied Biological Materials, Richmond, Canada) was used corresponding to manufacturer’s assignments.

### Microarray analysis

Biotinylated target cRNA was prepared as previously described [12]. A detailed protocol is available at www.affymetrix.com. Samples were hybridized to Affymetrix Human Gene 1.0 ST microarrays and analyzed by Affymetrix software expression console, version 1.1. For data analysis, robust multichip average (RMA) normalization was performed, including background correlation, quantile normalization, and median polish summary method. The volcano plot was generated using R (version 3.4.4; https://www.R-project.org/). To calculate *p*-values and log2 fold changes, the genes were tested for differential expression between groups based on log2 expression values. Before testing, all probe set IDs without mapped gene symbol were removed. The remaining 21,946 genes were tested for differential expression using the moderated t-statistic of the R package limma with default setting s. For gene ontology analysis overlapping up- or downregulated genes were identified using the jvenn platform [28] and analyzed via the Metascape platform with standard settings for pathway enrichment using GO Biological Processes, KEGG Pathway, Canonical Pathway and Reactome Gene Sets databases [29]. Array data in this manuscript were submitted at Gene Expression Omnibus (GEO) database, https://www.ncbi.nlm.nih.gov/geo/query/acc.cgi?acc=GSE162789.

### Proliferation assay

Cell proliferation was measured with an impedance-based instrument system (xCELLigence, Roche/ACEA Biosciences, Basel, Switzerland) enabling label-free real time cell analysis. Briefly, 2-4 × 10^4^ cells were seeded into 96-wells with 200 μl culture media containing FBS and allowed to grow up to 150 h. Cellular impedance was measured periodically every 4 h (relative cell index) and HDACi, EEDi, chemotherapeutics or DMSO was added as indicated in the Results section.

For synergy evaluation 0.5-1 × 10^4^ cells were seeded into white, flat-bottom, 96-well plates (Corning). Twenty-four hours later, doxorubicin and/or HDACi (FK228 or MS-275) were added, and viability was assessed after 72 h. Cell viability was measured using CellTiter-Glo Luminescent Reagent according to the manufacturer’s protocol (Promega). Luminescence was measured on a Promega GlowMax-Multi Detection System. Synergistic interactions were evaluated using the package Synergyfinder [30] (Package version 2.2.4, R version 4.0.3, RStudio version 1.3.1093) according to the Bliss algorithm [31].

### Colony forming assay

Cells were seeded in duplicate into a 35-mm plate at a density of 5 × 10^3^ cells per 1.5 ml methylcellulose-based media (R&D Systems, Minneapolis, MN, USA) according to the manufacturer’s instructions and cultured for 10-14 days at 37 °C/5% CO_2_ in a humidified atmosphere.

### In vitro invasion assay

To study cell invasion, the BioCoat™ Angiogenesis System: Endothelial Cell invasion was used (BD Biosciences, San Jose, CA, USA) according to the manufacturer’s instructions as described previously [32].

### Co-immunoprecipitation (Co-IP)

For Co-IP cells were seeded at 6-10 × 10^6^ per cell culture dish and cultivated until 70% confluence. Cells were subsequently harvested and treated using the Universal Magnetic Co-IP Kit (Active motif, Carlsbad, USA). Eluates were analyzed by western blotting.

### Western blot analysis

For western blots 2 × 10^6^ cells were resuspended in 200 μl 2x Laemmli Sample Buffer (0.125 M Tris HCl, 20% glycerol, 4% SDS and 0.002% Bromphenol Blue). Lysates were homogenized through a 23 gauge needle and denatured at 70 °C for 10 min. Between 18 and 25 μl of protein lysate was loaded on 12% SDS-PAGE and transferred onto a PVDF membrane (GE Healthcare Amersham™ Hybond™). After incubation with the specific primary antibody (see Supplementary Information), the membranes were incubated with appropriate secondary peroxidase-conjugated antibodies. For detection ECL™ Prime Western Blotting System (GE Healthcare, Chicago, IL, USA) and the Gel Logic 1500 imaging system analyzed with Kodak Molecular Imaging Software (Version 5.0) was used.

### Animal model

Immune deficient Rag2^−/−^γc^−/−^ mice on a BALB/c background were obtained from the Central Institute for Experimental Animals (Kawasaki, Japan) and maintained in our animal facility under pathogen-free conditions in accordance with the institutional guidelines and approval by local authorities (55.2.1-54-5232-42-2016). Experiments were performed in 6-20 week-old mice.

### In vivo experiments

To examine in vivo tumorigenicity of HDAC1 or HDAC2 CRISPR/Cas9 knockouts and their respective controls, 2-3 × 10^6^ EwS cells were injected subcutaneously into immune deficient Rag2^−/−^γc^−/−^ mice and xenograft tumor growth was monitored. Mice were investigated daily, tumor xenografts were measured with digital calipers, and tumor volume was calculated as (L x W^2^) / 2, where L is length and W is width. At the experimental endpoints, determined by completion of treatment or attainment of tumor burden exceeding 1cm^3^, mice were humanely euthanized, tumors excised and characterized.

To examine therapeutic value of HDACi Romidepsin (FK228) and EEDi (A-395) in vivo, 2-3 × 10^6^ EwS cells were injected subcutaneously into immune deficient Rag2^−/−^γc^−/−^ mice. Once tumor was palpable mice were divided randomly into four groups and treated either with 250 mg/kg body weight A-395, 2 mg/kg FK228 or A-395 in combination with FK228 or solvent controls. FK228 was administered intra peritoneal (i.p.) once per week, A-395 subcutaneous (s.c) twice a week for 4 weeks.

### Histology

Histological analysis of tumor specimens was performed in five tumors per group. Tissues were fixed in 10% neutral-buffered formalin solution for minimally 48 h, dehydrated under standard conditions (Leica ASP300S, Wetzlar, Germany) and embedded in paraffin. Serial 2 μm sections were prepared with a rotary microtome (HM355S, ThermoFisher Scientific, Waltham, USA) and subjected to histological and immunohistochemical analysis. Hematoxylin-Eosin (H.-E.) staining was performed on deparaffinized sections with Eosin and Mayer’s Haemalaun according to a standard protocol. Immunohistochemistry was performed using a Bond RXm system (Leica, Wetzlar, Germany, all reagents from Leica) with a primary antibody against cleaved caspase 3 (clone ASP175, 1:150, Cell Signaling 9664). All slides were scanned with a high-throughput scanning system (AT2, Leica). The percentage of tumor area occupied by cleaved caspase 3 positive tumor cells and the average number of mitoses per 10 high power fields (MI) in tumor cells was evaluated in all sections by experienced pathologists (TG/KS), and representative images were taken using Aperio ImageScope × 64 (version 12.4.0.7018, Leica).

### Statistical analysis

Data are mean ± SEM as indicated. Statistical analysis was performed by unpaired two-tailed Student’s *t*-test or log-rank test, using Prism7 (GraphPad, San Diego, CA, USA) or Excel (Microsoft, Redmond, WA, USA). *P*-values < 0.05 were considered statistically significant. Either exact *p*-values or *p < 0.05, **p < 0.005, ***p < 0.0005 were indicated. Volcano plots were drawn with the free software environment R (http://www.r-project.org/).

## Results

### Expression of HDAC class I genes in EwS

**Fig. 1:**
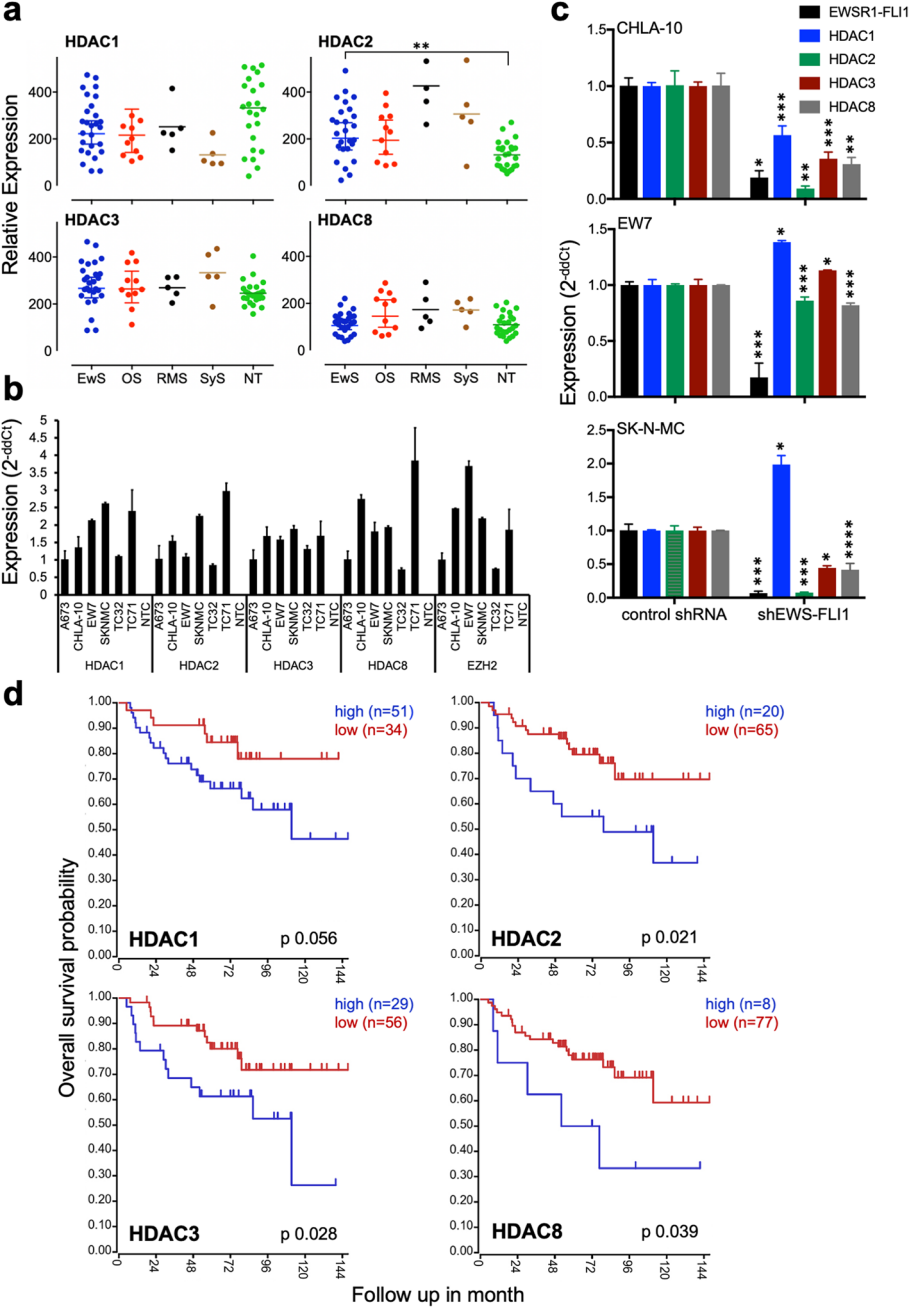
Expression of HDAC class I genes in EwS. **a** Gene expression profiles of HDAC class I genes *HDAC1, HDAC2, HDAC3, HDAC8* on 27 EwS, 11 osteosarcomas (OS), 5 rhabdomyosarcomas (RMS), 5 synovial sarcomas (SyS), and 25 samples of different normal tissue (NT). Patient RNA were hybridized onto Human Gene ST 1.0 arrays (Affymetrix; GSE45544, GSE73166) and compared to a published microarray study of normal tissue (GSE45544). **b** Expression of *class I HDAC* genes in different EwS cell lines determined by qRT-PCR. **c** Expression of *HDAC class I* genes are downregulated in EwS cell lines after transient shRNA transfection with EWS-FLi1 specific shRNA (see Materials and methods) measured by qRT-PCR. Control transfectants contained irrelevant shRNAs. Transfectants were treated with doxycycline for 72 h. **d** Low *HDAC class I* gene expression correlates with event-free survival: Kaplan-Meier estimates for event-free survival probability for HDAC class I gene expression, using the GSE63157 study set (*n* = 85) and the amc onco-genomics software tool (https://hgserver1.amc.nl/cgi-bin/r2/main.cgi)

Histone deacetylase (HDAC) class I genes *HDAC1, 2, 3,* and *8* are widely expressed in different pediatric sarcomas (Fig. 1a) and other pediatric and adult tumor entities (Additional file 2: Fig. S1a). Interestingly, the local tumor site in EwS may influence its level of expression (Additional file 2: Fig. S1b). In EwS cell lines *HDAC class I* genes are well expressed, too (Fig. 1b). To analyze whether the oncogenic fusion protein EWS-FLI1 may influence *HDAC class I* gene expression in EwS, we investigated RNA interference-mediated EWS-FLI1 silencing and observed a significant suppression of HDAC class I expression levels in two different EwS cell lines while one (EW7) seemed less affected (Fig. 1c). However, overexpression of EWS-FLI1 in mesenchymal stem cells L87 and V54.2 [12] only resulted in an upregulation of HDAC3 and 8 (Additional file 2: Fig. S1c). Remarkably, we also observed with a varying degree of significance that the level of individual class I HDAC expression may be associated with event-free survival in EwS when analyzing a publicly available study set of 85 patients (GSE63157, Fig. 1d).

### Influence of HDAC inhibitors on histone modification and expression profile

**Fig. 2:**
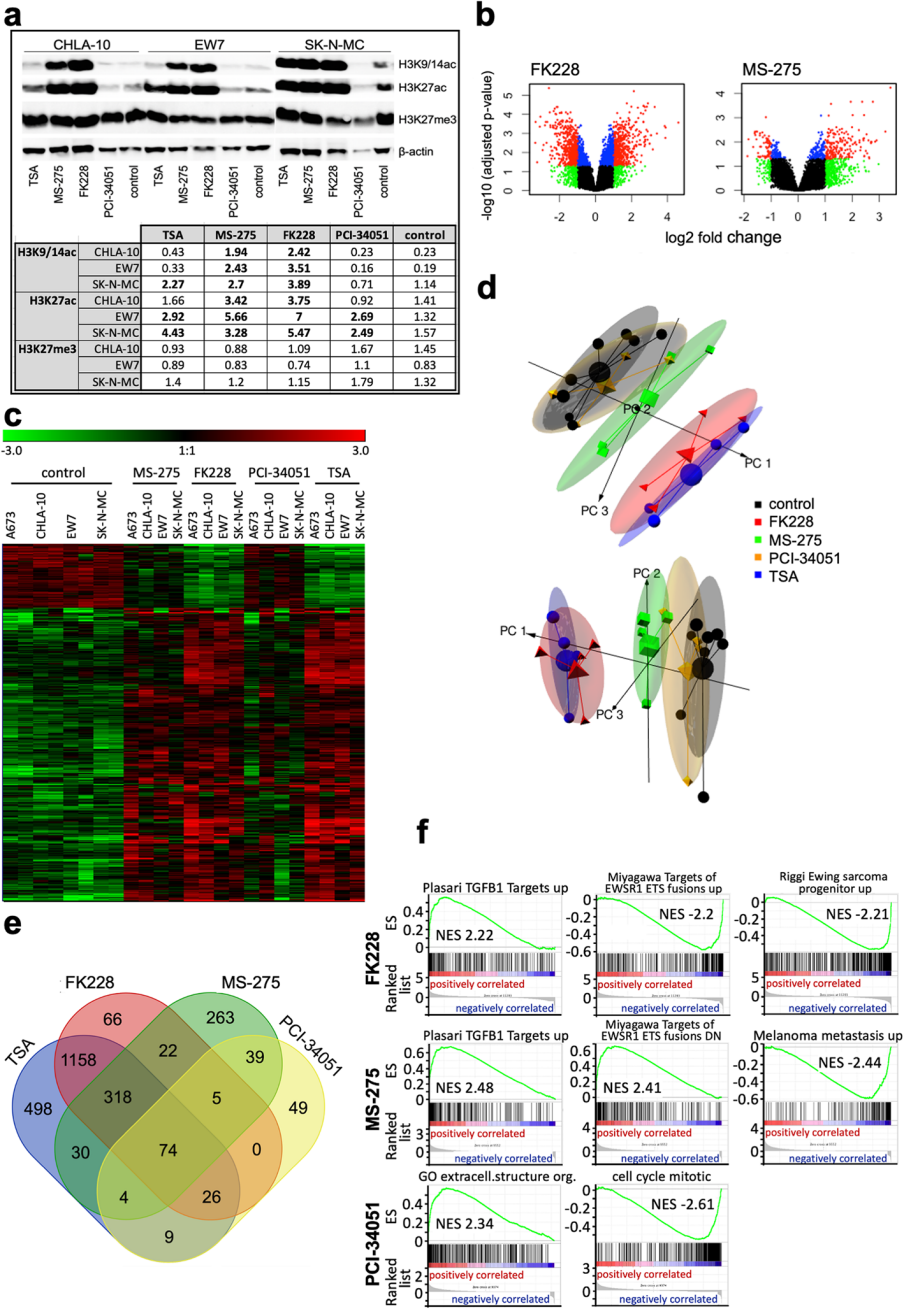
Influence of HDAC inhibitors on histone modification and expression profile. **a** (top) WB analysis of H3K9/14 ac, H3K27ac, H3K27me3 after HDACi treatment or solvent control (DMSO) for 17 h of three different EwS lines. β-actin served as loading control. (bottom) a table of the optical density of the individual band signals is given as the quotient of the individual value and the control (ß-actin). Values > 1.8 are shown in bold. **b** Volcano plots of differentially expressed genes averaged across four EwS lines (A673, CHLA-10, EW7, SK-N-MC). Each plot shows fold changes of log2 expression values (log FC) and adjusted *p*-values obtained from differential expression analysis comparing cell lines treated with HDACi (FK228 or MS-275, respectively) to solvent controls*.* Red, genes obtained adjusted *p*-value < 0.05 and absolute log FC > 1; blue, genes with adjusted *p*-value < 0.05 and absolute log FC ≤ 1; green, genes with adjusted *p*-value ≥0.05 and absolute log FC > 1; and in black, genes with adjusted *p*-value ≥0.05 and absolute log FC ≤ 1. **c** Heat map of 476 genes, 2-fold differentially expressed after HDACi treatment in four different EwS lines. Each column represents one individual array (robust multichip average (RMA); GSE162785). Cells were treated with DMSO or HDACi for 17-30 h. **d** Principal Component Analysis (PCA) plot showing the Principal Components (PC) 1 to 3. A dot represents one sample of the four different EwS lines, each treated with four HDACi, respectively (FK228, MS-275, PCI-34051, TSA). Two replicates of each cell line are shown as controls. In each cluster, a large dot in the center represents the average. Both PCA plots depict the same data from different perspectives. **e** Shared genes differentially expressed after individual treatment with different HDACi in four EwS lines (see [18]; GSE162785), respectively. For Venn diagram, genes ±2-fold differentially expressed were selected for analysis (http://bioinformatics.psb.ugent.be/webtools/Venn/). **f** GSEA enrichment plots of up- and downregulated gene sets after individual HDACi treatment. NES: normalized enrichment score. GSEA: http://www.broadinstitute.org/gsea/index.jsp

To further investigate the role of class I HDACs in EwS we first used individual HDAC inhibitors (HDACi) to determine their role in histone modifications and subsequently monitored their influence on the general expression profile in EwS cell lines. To assess global alterations of histones (H3K9/14-acetylation, H3K27-acetylation, H3K27-trimethlation) in EwS cell lines, tumor cells were incubated for 17 h with different HDACi. Western blot analysis showed an increase of histone H3K27-acetylation and H3K9/14-acetylation after HDACi treatment in all cell lines (Fig. 2a). An even higher increase of the acetylation of H3K27 and H3K9/14 was observed after treatment with Entinostat (MS-275) or Romidepsin (FK228) in CHLA-10 and EW7 cells. In contrast, treatment with PCI-34051 only resulted in some increase of H3K27ac in comparison to the control. As expected, no alterations of H3K27-trimethylation were seen after treatment with HDACi.

HDACi-induced changes in gene expression in EwS cells A673, CHLA-10, EW7 and SK-N-MC were investigated by microarray analysis. Differentially expressed genes averaged across the used EwS cell lines are visualized in volcano plots after treatment with Romidepsin or Entinostat (Fig. 2b). Significant deregulated genes are depicted in red. Treatment with Romidepsin led to differential expression of 2312 genes, treatment with Entinostat led to differential expression of 475 genes, respectively. Differential expression analysis comparing four different HDACi at a fold change of ±2 identified 390 up- and 86 downregulated genes (Fig. 2c). Principal component analysis demonstrated that the level of differential gene expression, mediated by individual HDACi treatment, led to the strongest deregulation of the EwS expression profile using TSA and Romidepsin (Fig. 2d). Using venn diagram analysis the strongest overlap of deregulated genes was observed after TSA and Romidepsin treatment (Fig. 2e). Furthermore, gene set enrichment analysis (GSEA) enrichment plots of up- and downregulated gene sets after individual HDACi treatment showed that both Entinostat and Romidepsin treatment induced gene sets important for differentiation and downregulated gene sets important for an EwS specific expression profile. Entinostat in addition, reduced gene sets important for metastasis. However, PCI-34051 deregulated lesser gene sets, but those important for cell growth and survival (Fig. 2f).

The EwS specific inhibition of differentiation by class I HDACs poses the question whether treatment with Entinostat or Romidepsin would increase the ability to induce differentiation of EwS cells. We could observe a clear increased formation of endothelial differentiation (Additional file 2: Fig. S2a) and enhanced expression of *GAP43* and *GFAP* as markers for neuronal differentiation (Additional file 2: Fig. S2b) after treatment with one of the two inhibitors.

Overall, these results demonstrate that e.g. more specific HDAC class I inhibitors such as Entinostat (MS-275; HDAC1 and 3 inhibitor) and Romidepsin (FK228; HDAC1 and 2 inhibitor) strongly deregulate the EwS specific profile and induce differentiation, with Romidepsin showing comparable results to the pan HDACi TSA.

### Influence of CRISPR/Cas9 HDAC knockouts on proliferation and invasiveness

**Fig. 3:**
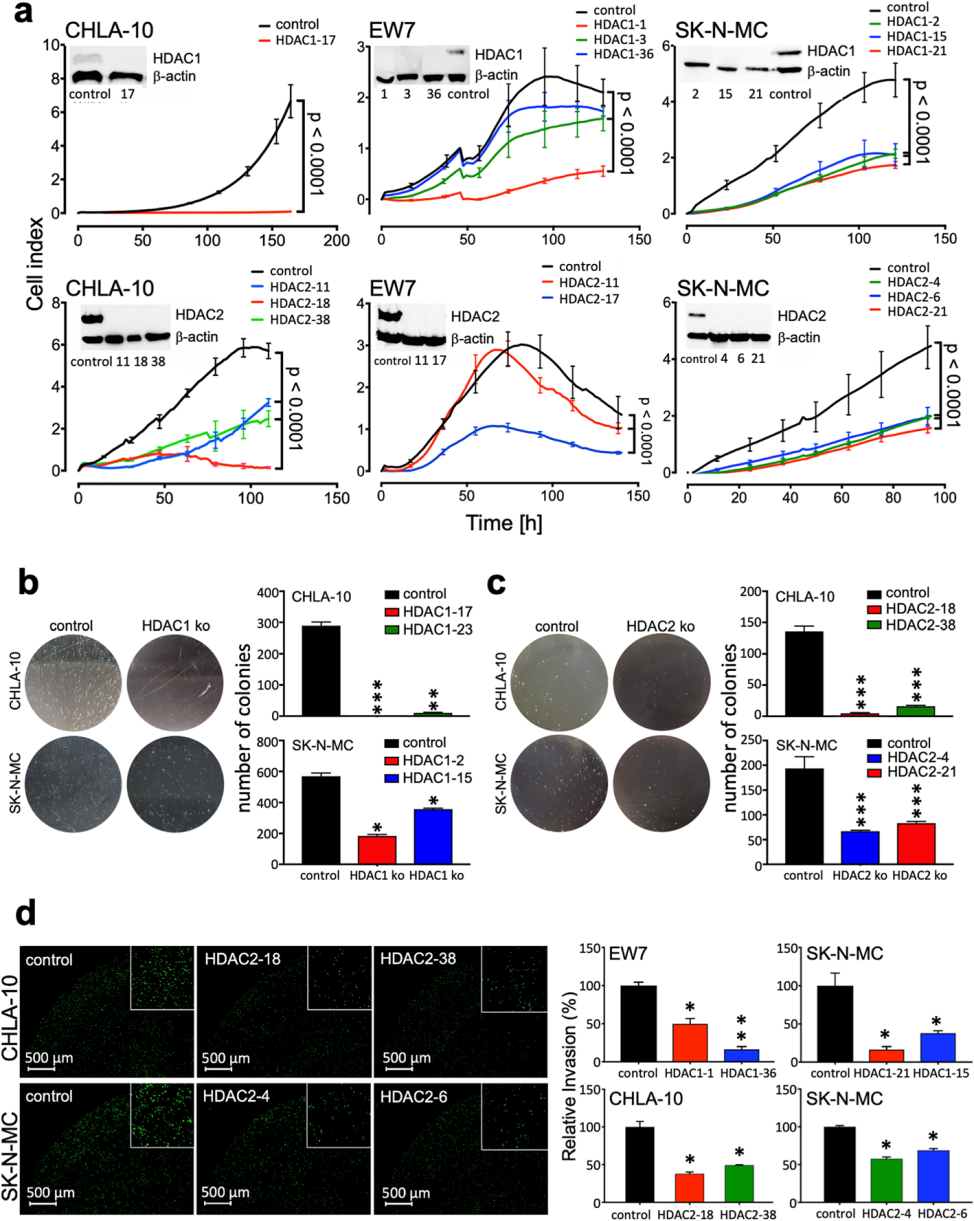
Influence of CRISPR/Cas9 HDAC knockouts on proliferation and invasiveness. **a** Proliferation and Western blot analysis (insets) of one to three HDAC1 (top) or HDAC2 (bottom) CRISPR/Cas9 knockouts in EwS cell lines CHLA-10, EW7 and SK-N-MC in comparison to controls (Cas9) are shown. Protein levels of Western blot analysis were detected with antibodies against HDAC1 and HDAC2. β-actin antibodies served as loading control. Proliferation and cell impendence was measured by the xCELLigence assay every 4 h. Data are shown as mean ± SEM (hexaplicates/group; *p*-value < 0.0001). **b** and **c** Analysis of anchorage-independent colony formation in methylcellulose of EwS cell lines with CRISPR/Cas9 mediated knockout of HDAC1 or HDAC2. Macrographs show a representative experiment with CHLA-10 and SK-N-MC HDAC1 ko (**b**) or HDAC2 ko (**c**). The number of colonies in different CHLA-10 or SK-N-MC HDAC clones was summarized in two bar graphs. **d** Analysis of invasiveness through Matrigel of different HDAC1 and HDAC2 CRISPR/Cas9 knockouts. Left, representative micrographs of invasive HDAC2 knockout and control cells 48 h after incubation are shown. Right, quantitative results for two different EwS lines are shown. Data are mean ± SEM of two independent experiments

HDACi usually inhibit a group of HDAC genes, even those more specific class I HDACi. To find out which role individual HDACs may play in the pathogenesis of EwS, the CRISPR/Cas9 method was used to selectively knockout HDAC1 or HDAC2 in different EwS cell lines. The detection of HDAC class I knockouts in EwS cell lines was done by western blot analysis (Fig. 3a). Proliferation with the xCELLigence system demonstrates a significant decreased proliferation ability of HDAC1 and HDAC2 knockouts in EwS cell lines in comparison to their control (Fig. 3a).

To investigate whether a decreased growth rate of these HDAC knockouts also affect their phases of cell cycle we used flow cytometry. We observed extended G1 phases for HDAC1 knockouts, especially in SK-N-MC, EW7 and less evident in CHLA-10 cells (Additional file 2: Fig. S3a). For HDAC2 knockouts we saw a tendency for an extended G2 phase in all three cell lines tested (Additional file 2: Fig. S3a). We also in some HDAC knockouts, especially in CHLA-10 and SK-N-MC, saw an increase of sub G1, indicating a higher rate of apoptosis. This prompted us to use immunocytochemistry of γ-H2AX for these HDAC1 and HDAC2 knockouts and indeed the investigated EwS cell lines show significant more double-strand breaks than their controls (Additional file 2: Fig. S3b).

These findings correlate with results of the analysis of anchorage-independent growth of CRISPR/Cas9 knockouts of HDAC1 or HDAC2 which demonstrated their critical contribution to colony formation ability in EwS cells (Fig. 3b, c). Furthermore, in vitro invasiveness was similarly affected. HDAC1 and 2 knockouts had a decreased potential for invasiveness as demonstrated by Matrigel assay (Fig. 3d).

To further investigate if individual HDACs may counterbalance knockouts of the other we inspected individual class I HDAC expression in HDAC knockouts and their controls by western blot analysis. We clearly observed increased HDAC2, 3, and even 8 expression in HDAC1 knockouts at the protein level indicating compensatory mechanisms (Additional file 2: Fig. S4a, top). However, the results for HDAC2 knockouts were less clear, but there were some compensatory effects for HDAC3 and 8, too (Additional file 2: Fig. S4a, bottom). In addition, microarray analysis of HDAC knockouts revealed less deregulated genes when compared to treatment of EwS cells with HDACi Entinostat or Romidepsin. Again, this indicates compensatory mechanisms of other class I HDACs in these knockout cells (Additional file 2: Fig. S4b, c).

Various attempts to generate stable HDAC3 CRISPR/Cas9 knockouts in EwS cell lines were not successful, suggesting HDAC3 to be essential for survival; while sgRNAs used for HDAC8 CRISPR/Cas9 knockouts seemed not effective. Therefore, the role of HDAC3 and 8 was further investigated by transduction with inducible shRNA constructs (Additional file 2: Fig. S5a). After downregulation of HDAC3 or HDAC8 in EwS cell lines, proliferation assay showed only a slight difference in proliferation ability for HDAC3 knock downs (Additional file 2: Fig. S5b above) and even less difference for HDAC8 knock downs compared to their control (Additional file 2: Fig. S5b at the bottom left). However, HDAC8 downregulation in SK-N-MC HDAC1 knockouts showed a stronger block of proliferation than HDAC1 knockouts without HDAC8 downregulation (Additional file 2: Fig. S5b at the bottom right). In addition, HDAC3 knock down resulted in reduced invasiveness of CHLA-10 cells while similar HDAC8 downregulation in SK-N-MC HDAC1 knockouts demonstrated a superior reduction in invasiveness than without HDAC8 downregulation (Additional file 2: Fig. S5c).

### HDAC1 and HDAC2 increase local tumor growth in vivo

**Fig. 4:**
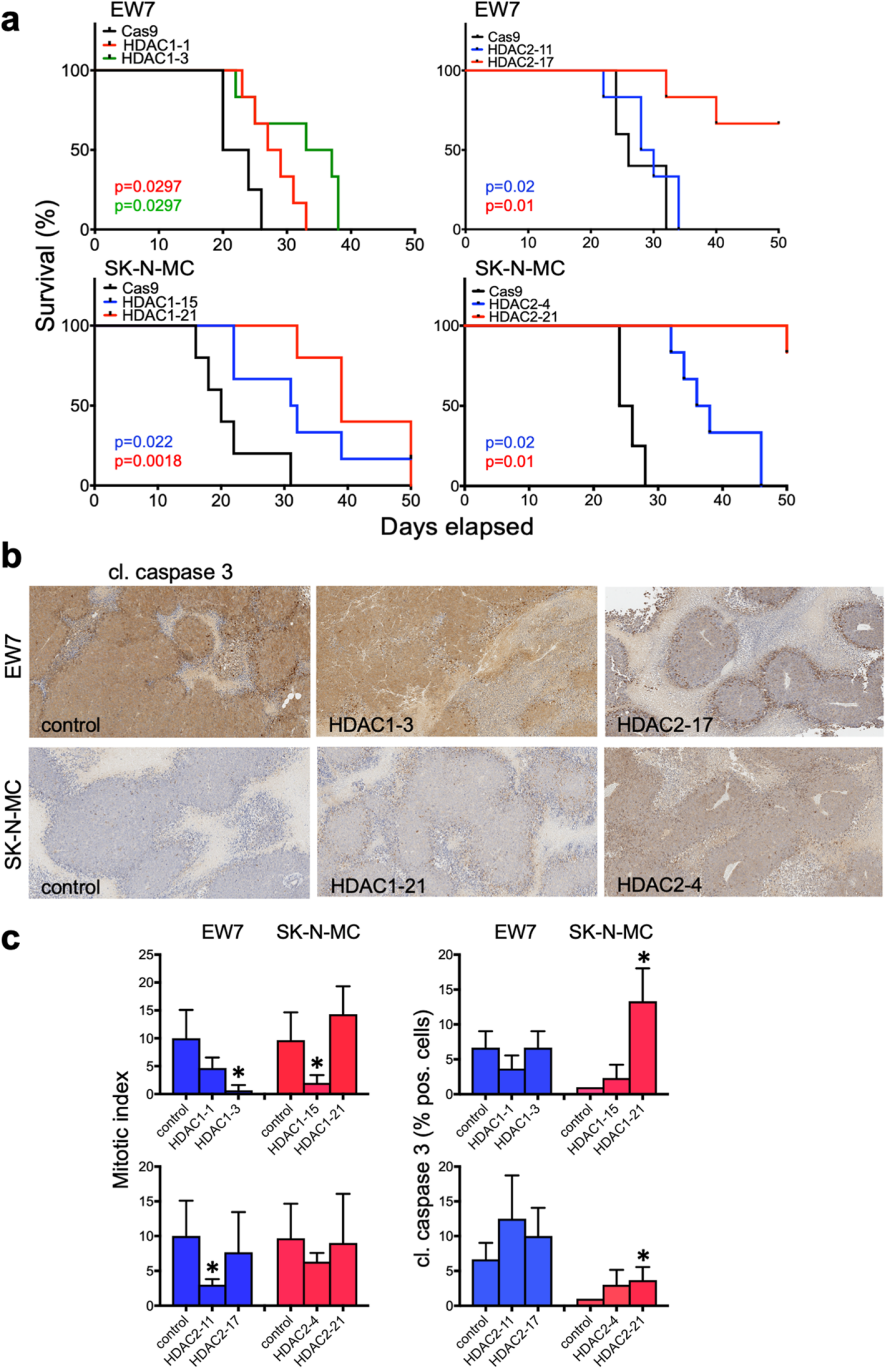
HDAC1 or HDAC2 increases local tumor growth in vivo. Evaluation of tumorigenicity of CRISPR/Cas9 knockouts of HDAC1 or HDAC2 and their controls (Cas9) in EwS cell lines EW7 and SK-N-MC. **a** Immune deficient Rag2^−/−^γ_C_^−/−^mice were injected s.c. with 4 × 10^6^ EwS cells. Kaplan-Meier plots of individual experiments with four to six mice per group are shown. Log-rank test was used to test for differences in survival. **b** Tumors of sacrificed mice were analyzed for cleaved caspase 3 by immunohistochemistry. Representative results of EW7 and SK-N-MC tumors are shown (10x original magnification). **c** Left, the number of mitoses per 10 high power fields per tumor is given. Right, level of cleaved caspase 3 in EwS tumors. The percentage of cleaved caspase 3 positive cells in five fields per tumor is given

To assess whether HDAC1 or HDAC2 knockouts also show a reduced tumor formation ability in vivo, local tumor growth of CRISPR/Cas9 knockouts of HDAC1 or HDAC2 in EwS cell lines EW7 and SK-N-MC was analyzed. In a xenograft mouse model HDAC1 and HDAC2 knockouts demonstrated reduced local tumor growth in all cell lines (Fig. 4a; and CHLA-10, see Additional file 2: Fig. S5d), resembling their proliferation ability in vitro (Fig. 3a). Immunohistochemistry of HDAC knockout tumors and their controls demonstrated an increased cleaved caspase 3 expression in some HDAC knockouts, irrespective whether HDAC1 or HDAC2 knockouts were monitored (Fig. 4b, c right). However, increased caspase 3 cleavage was not a general phenomenon in these knockout cells. In addition, analysis of the mitotic index revealed decreased mitosis for some knockouts in comparison to their controls (Fig. 4c left), but not for all of them. This argues for a mixture of events explaining decreased tumor growth of HDAC1 and HDAC2 knockouts.

### HDAC inhibition increases susceptibility to first line chemotherapy

**Fig. 5:**
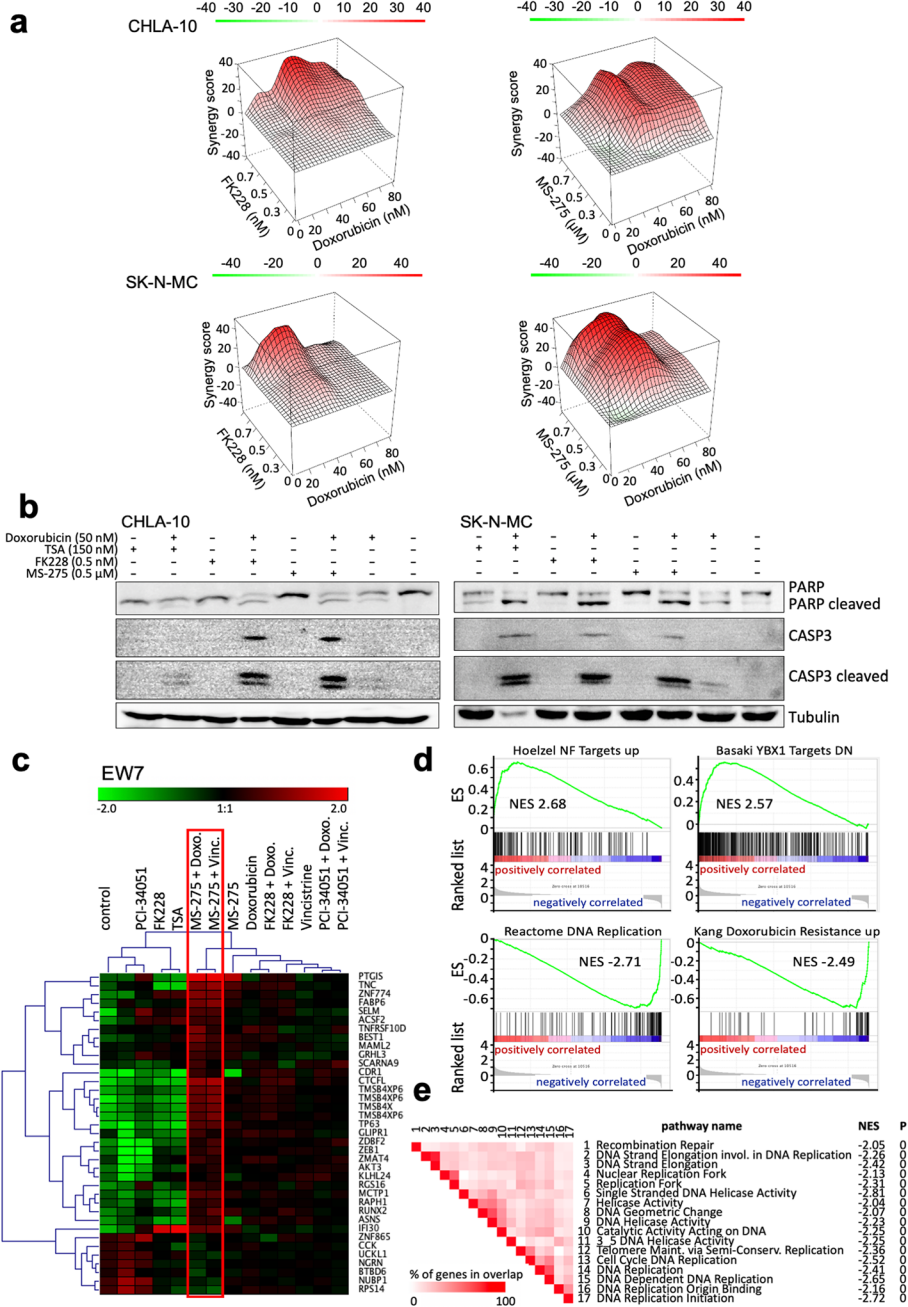
HDAC inhibitors increase susceptibility of EwS to first line chemotherapeutics. **a** Heatmaps showing excess over Bliss synergy scores for combination treatment of Doxorubicin with HDACi FK228 (left) or MS-275 (right) in CHLA-10 or SK-N-MC cells. Scores greater than 1 (shown in red) denote synergistic combinations, whereas scores less than 1 (shown in green) denote antagonistic combinations. Data represent 3 technical triplicates. **b** Western blot analysis of caspase 3 and PARP as a measure of apoptosis susceptibility after treatment with Doxorubicin and HDACi, either in combination or individually. CHLA-10 were treated for 36 h and SK-N-MC for 24 h. Tubulin was used as loading control. **c** Focused heat map of genes, ≥1.5-fold differentially expressed in EwS line EW7 after combined treatment with DMSO, HDACi and/or first line chemotherapeutics Doxorubicin or Vincristine for 17 h. Treatment with Doxorubicin or Vincristine and MS-275, respectively, are highlighted in red. Each column represents one individual array. Microarray data with their normalized fluorescent signal intensities were used (robust multichip average (RMA); GSE162785). **d** GSEA enrichment plots of up- and downregulated gene sets after combined treatment with MS-275 and Doxorubicin. NES: normalized enrichment score. GSEA: http://www.broadinstitute.org/gsea/index.jsp. **e** GSEA leading-edge analysis of identified gene sets (C5_all, GO gene sets) downregulated after combined treatment with MS-275 and Doxorubicin. Set-to-set analysis shows a correlation between combined MS-275 and Doxorubicin treatment and downregulation of gene sets important for chromosomal regulation

Results so far indicated that HDAC1, HDAC2 and in part HDAC3 are important mediators of the EwS typical expression profile and malignant phenotype. Nevertheless, monotherapy using HDACi so far was not proven successful in EwS [33]. Therefore, we asked, whether HDACi may increase susceptibility of EwS to other drugs. Doxorubicin and Vincristine are first line chemotherapeutic agents used in the antitumor therapy of EwS patients [34]. For Doxorubicin and HDACi combination therapy in comparison to Doxorubicin monotherapy, a significant increase in proliferation blockade and an increase in apoptosis was observed (Additional file 2: Fig. S6). Combination index analysis of the combined effect of these drugs on proliferation (see Material and methods) using the Bliss algorithm [30] revealed a good synergy score for all combinations tested (Fig. 5a). Furthermore, western blot analysis of cleaved caspase 3 and PARP expression in EwS cell lines treated with Doxorubicin and HDACi demonstrated a strong induction of apoptosis after receiving combined treatment (Fig. 5b).

Expression analysis after combined treatment of Entinostat and Doxorubicin revealed increased pro-apoptotic gene expression, ubiquitin-mediated protein degradation and AKT signaling (Fig. 5c, d) combined with decreased S-phase activity and resistance to Doxorubicin (Fig. 5d, e).

Furthermore, HDAC1 knockouts in EwS cells CHLA-10 and SK-N-MC are significantly more susceptible to chemotherapeutics such as Vincristine, than their control (Additional file 2: Fig. S7a top). But combined treatment of parental EwS cells with HDACi Entinostat or Romidepsin in combination with Vincristine did not reveal synergistic treatment effects (Additional file 2: Fig. S7a, bottom). However, gene expression analysis after treatment with Romidepsin, Vincristine and a combination of both identified relevant gene up- and downregulation only present in the combination therapy, such as decreased double strand break repair (Additional file 2: Fig. S7b).

### Combined inhibition of PRC2 and class I HDAC synergize to block proliferation and tumor growth

**Fig. 6:**
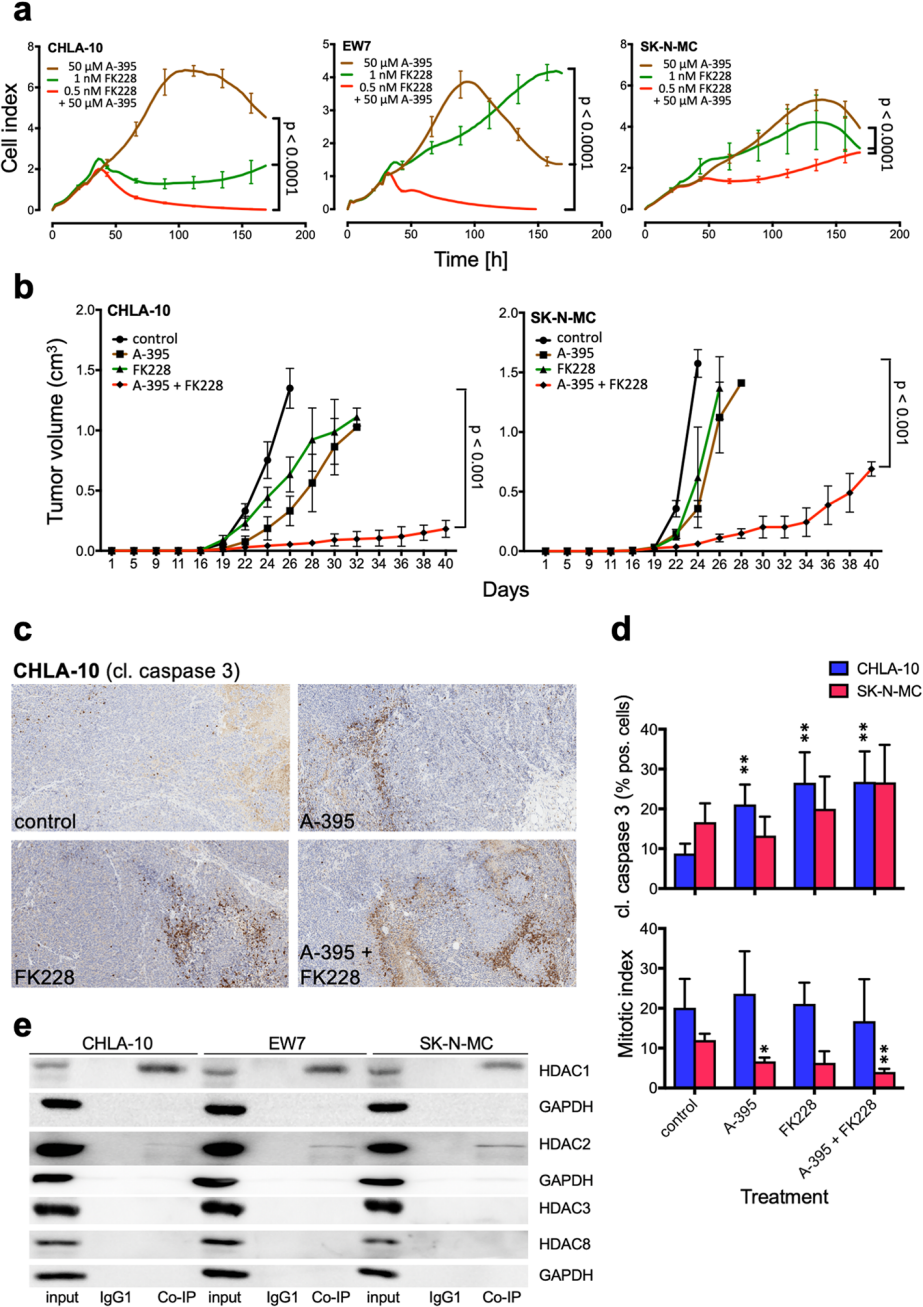
Combined targeting of PRC2 and class I HDACs results in the inhibition of proliferation and tumor growth. **a** Proliferation analysis of EwS cell lines CHLA-10, EW7 and SK-N-MC treated with either A-395, FK228 or a combination of both. Cell impedance was measured every 4 h. Data are shown as mean ± SEM (hexaplicates/group; *p*-value < 0.0001). **b** In vivo antitumor efficacy of FK228 and A-395 as a single agent or in combination against tumor-bearing Rag2^−/−^γ_C_^−/−^mice. 2 × 10^6^ CHLA-10 or SK-N-MC EwS cells were injected subcutaneously (s.c.) into mice. Once tumors were palpable mice were randomly allocated into four groups (5 mice/group) and treated with vehicle control (DMSO), A-395 (250 mg/kg, s.c. twice per week), FK228 (2 mg/kg, intraperitoneal (i.p.) once per week), or with A-395 in combination with FK228 for 4 weeks (*p* < 0.0001). **c** All tumors were analyzed for cleaved caspase 3 by immunohistochemistry. Representative results of CHLA-10 tumors are shown (10x original magnification). **d** Top, the level of cleaved caspase 3 in CHLA-10 and SK-N-MC derived tumors. The percentage of cleaved caspase 3 positive cells in five fields per tumor is given. Bottom, the number of mitoses per 10 high power fields per tumor is given. **e** Interaction analysis by Co-IP of PRC2 protein EED with HDAC1, and HDAC2. Co-IP for CHLA-10, EW7, and SK-N-MC cell lines using anti-EED antibodies. After Co-IP, proteins were analyzed by western blotting for HDAC1, 2, 3 and 8. GAPDH served as a loading control

Histone methyltransferase EZH2 together with SUZ12 and EED are essential components of PRC2. Due to the findings that class I HDACi inhibits an EWS-FLI1 specific expression profile and knockouts of HDAC1 and HDAC2 blocks tumorigenicity, which is comparable to RNA interference of EZH2 [12], we tested whether combined treatment of HDACi together with PRC2 blockade may synergize in vivo and in vitro. Here we chose to use an EED inhibitor (A-395) and combined it with Romidepsin. Proliferation assay of EwS cell lines CHLA-10, EW7 and SK-N-MC showed a significant inhibition of proliferation after treatment with a combination of HDACi and EEDi, demonstrating superiority to monotherapy (Fig. 6a). Combined inhibition of spheroid cell growth of EwS cell lines and an EwS PDX derived primary culture was similarly effective (Additional file 2: Fig. S7c). In addition, similar synergistic behavior of combined therapy with HDACi and EEDi was also observed in vivo. Tumors of SK-N-MC or EW7 in xenograft mice showed a strong inhibition of local tumor growth after treatment with HDACi and EEDi (Fig. 6b). Of note, single high doses of EEDi were ineffective in vitro and in vivo (Fig. 6a, b).

Western blot analysis of apoptosis susceptibility after Romidepsin or Entinostat and/or A-395 treatment in vitro demonstrated a significant caspase 3 and PARP cleavage only after combined treatment (Additional file 2: Fig. S8a), respectively. Furthermore, immunohistochemistry of treated tumors and their controls also indicated an increased cleaved caspase 3 expression in treated tumors, irrespective whether CHLA-10 or SK-N-MC tumors were analyzed (Fig. 6c, d top). Enhanced apoptosis seemed strongest after combined treatment, however, this was not statistically significant. Furthermore, analysis of the mitotic index revealed a tendency for decreased mitosis in SK-N-MC tumors after HDACi and/or EEDi treatment in comparison to their controls (Fig. 6d bottom).

Expression analysis of treated tumors identified a number of differentially expressed genes including decreased expression of pediatric cancer markers and EZH2 targets only after combined treatment (Additional file 2: Fig. S8b, c, d). Final Co-IP experiments in EwS cells confirmed an interaction of PRC2 together with HDAC1 and 2 in EW7 and SK-N-MC cells, but not with HDAC3 and 8 (Fig. 6e).

## Discussion

The expression of EWS-FLI1 in EwS leads to large-scale epigenetic alterations that result in global modifications of histone H3K27-acetylation as well as H3K27-trimethylation in conjunction with altered histone deacetylase (HDAC) activity [10, 11]. *HDAC class I* genes are strongly expressed in various pediatric sarcomas and other pediatric and adult tumor entities. EwS are very susceptible to HDAC inhibition [12, 23–25]. The activity of e.g. Entinostat in EwS seems to be mediated by the inhibition of DNA synthesis, cell cycle arrest, the increase of expression of p21, TGF-βRII and c-myc and the induction of apoptosis [23, 24]. Our western blot analyses revealed increased histone H3K27-acetylation and H3K9/14-acetylation not only after treatment with the pan-HDACi TSA, but also with more class I specific HDACi Entinostat (MS-275) or Romidepsin (FK228). However, treatment with PCI-34051 did not result in histone changes, suggesting that HDAC8 is not involved in these processes.

Subsequent gene expression analysis after individual class I specific HDACi treatment showed induction of genes important for cellular differentiation and a strong inhibition of EwS-specific expression profiles by Entinostat or Romidepsin. PCI-34051 deregulated less individual genes in EwS, but those important for cell growth and survival. These observations support previous results demonstrating that Romidepsin decreased EWS-FLI1 mRNA levels [35], and that Romidepsin and Entinostat induced antiproliferative activity in EwS cells [24, 35].

In addition, individual treatment with either Entinostat or Romidepsin resulted not only in an increased expression of genes relevant for cellular differentiation, but also in an increased ability of EwS cells for endothelial or neuronal differentiation. These observations correlate with previous results demonstrating that class I HDAC support an EwS-specific malignant stemness expression profile [12]. This also partly explains our observation that a high individual class I HDAC expression correlate with poor prognosis in EwS patients. Similar results were observed in other tumor entities. Increased expression of HDAC1, 2 and 3 has been associated with poor outcome in gastric and ovarian cancer [36–38], while high HDAC8 levels correlate with advanced disease and poor survival in neuroblastoma [39, 40].

Genetic analysis of the role of individual HDAC class I genes by CRISPR/Cas9 knockouts showed that both HDAC1 and 2 expression are essential for proliferation, invasiveness and local tumor growth of EwS cells. In addition, prolonged sub-G1 phases in cell cycle analysis in some HDAC1 and 2 knockouts as well as increased γ-H2AX signals, indicate an increased rate of apoptosis and significant increase of double-strand breaks, respectively.

Immunohistochemistry of EwS tumors revealed an increased expression of cleaved caspase 3 in some HDAC knockouts, regardless of whether HDAC1 or HDAC2 knockouts were analyzed. This suggests that knockouts of these genes in EwS are associated with a certain cellular stress. This hypothesis is supported by the observation that especially in HDAC1 knockouts an increased expression of HDAC2, HDAC3 and even HDAC8 was observed in western blot analysis, indicating compensatory mechanisms by other HDAC of this class. However, for HDAC2 knockouts only low compensatory expression of other HDAC genes was witnessed. HDAC1 and 2 are often co-expressed to jointly regulate many biological processes, such as DNA damage repair, cell cycle, autophagy and hematopoiesis [41]. Interestingly, depletion or inhibition of HDAC1 and 2 in primary or oncogene-transformed fibroblasts resulted in a senescence-like G1 cell cycle arrest, accompanied by upregulation of p21 and p53 [42].

Our attempts to generate stable HDAC3 CRISPR/Cas9 knockouts in EwS cell lines were not successful. We were only able to generate transient knockouts indicating that HDAC3 is essential for the survival of EwS cells. However, subsequent RNA interference analysis of the role of HDAC3 and 8 in EwS only revealed a small difference in proliferation ability for HDAC3 knock downs and even fewer differences for HDAC8 knock downs compared to their controls. Remarkably, HDAC8 downregulation in SK-N-MC HDAC1 knockouts demonstrated a stronger block of proliferation and reduction of invasiveness than without HDAC8 downregulation overall indicating additive effects in EwS when more than one HDAC member of this class is affected. HDAC3 and HDAC8 seem differentially regulated, not only in EwS. While HDAC3 associates with other proteins than HDAC1 and 2, for HDAC8 no protein complex has been described [22].

Our results suggest that HDAC1, 2 and partly HDAC3 are important mediators of the EwS-typical expression profile and the malignant stemness phenotype. However, monotherapy with HDACi so far was not successful. Generally, monotherapy with suberoylanilide hydroxamic acid (SAHA) an inhibitor of class I and II HDACs seems to be less effective in solid tumors [43]. In childhood cancer, phase I studies with SAHA alone and in combination with 13-cis-retinoic acid [44], Temozolomide [45] or Bortezomib [46] have been completed. A recent phase I study with Entinostat as a monotherapy to determine dosage and safety is being evaluated in pediatric patients with recurrent or refractory solid tumors (NCT02780804), including central nervous system (CNS) tumors and lymphomas, but not in EwS or other pediatric sarcoma patients. A study investigating SAHA together with backbone chemotherapy in pediatric patients with tumors including Ewing and rhabdomyosarcoma is recruiting (NCT04308330).

First line chemotherapeutic agents used in antitumor therapy of EwS patients include Doxorubicin and Etoposide [34]. They are both topoisomerase II enzyme inhibitors. SAHA antagonistically affected the antiproliferative effect of Doxorubicin in the majority Ewing sarcoma cells, but synergistically enhanced the antiproliferative activity of etoposide [33]. Here, HDACi treatment increased the susceptibility of EwS cells to Doxorubicin, irrespective whether suboptimal doses of Entinostat or Romidepsin were used. In combination suboptimal doses of Doxorubicin and HDACi significantly blocked growth of EwS cells and increased apoptosis compared to Doxorubicin monotherapy. Expression analysis of such combined treatment with Entinostat and Doxorubicin revealed an increased pro-apoptotic gene expression, ubiquitin-mediated protein degradation and AKT signaling in combination with decreased S-phase activity and resistance to Doxorubicin. HDACs seem essential for heterochromatin formation [47]. When such formation is inhibited, DNA would be structurally less dense and more open to agents that damage DNA and increase nucleosome turnover such as the anthracycline Doxorubicin [48].

Another very interesting option for combination therapy appeared a combination of HDACi with PRC2 inhibitors. We had already observed that HDACi class I inhibit an EWS-FLI1-specific expression profile and that the elimination of HDAC1 and HDAC2 reduces the tumor susceptibility of EwS cells. Interestingly, the analysis of expression profiles after RNA interference of EZH2 and HDACi treatment with TSA showed very similar expression patterns [12]. Therefore, a combination of an EED inhibitor (A-395) [49] together with Romidepsin seemed reasonable. Suboptimal doses of both substances that were effective only in their combination, synergistically suppressed proliferation in vitro as well as in vivo and induced significant caspase 3 or PARP cleavage. This combination therapy was similarly effective in the spheroid model of EwS cell lines and two primary cultures derived from EwS PDX. It is noteworthy that high doses of EEDi monotherapy had no effect on tumor proliferation. Expression analysis of treated tumors identified decreased expression of pediatric cancer markers and EZH2 targets, only after combined treatment, supported by Co-IP experiments confirming an interaction of PRC2 together with HDAC1 and 2 in EwS cells.

We had already observed in previous experiments that results after RNA interference of EZH2 [12] could not be reproduced with EZH2 inhibitors such as GSK126 or GSK343 (data not shown). An EED inhibitor appeared interesting because it not only suppressed EZH2 activity but also potentially inhibited the presence of PRC2 in chromatin [49]. The lack of effect of EZH2 inhibitors also suggested that HDACs interacting with PRC2 mediated the effects on the expression profile previously observed after RNA interference of EZH2 [12].

## Conclusions

HDAC1 and HDAC2 are important mediators of the pathognomonic EWS-ETS-mediated transcription program in EwS. Their knockout blocks proliferation, invasiveness and tumor growth in xenograft mice. Expression analyses of EwS cells demonstrated that treatment with individual HDACi blocked an EWS-FLI1-specific expression profile and increased susceptibility to treatment with first line chemotherapeutic agents such as Doxorubicin. Combination therapy of HDACi together with PRC2 inhibitors like the EED inihibitor A-395, that interfere with chromatin binding of the PRC2/HDAC1/2 complex, significantly block tumor growth of EwS in a xenograft mouse model and could be an interesting new treatment option for this malignant disease.

## Supplementary Information

**Additional file 1.** Supplementary Materials and methods.

**Additional file 2: Fig. S1 a**, Expression levels of different class I HDAC genes in different pediatric small-round-blue-cell tumors, carcinomas and normal tissues by box plot presentation using a comparative study of the amc onco-genomics software tool (https://hgserver1.amc.nl/cgi-bin/r2/main.cgi). The number of samples in each cohort is given in brackets. **b**, Differential expression levels of class I HDAC genes in primary EwS at different tumor sites by box plot presentation using the GSE63157 study set and the amc onco-genomics software tool. The number of samples in each cohort is given in brackets, ND: not determined. *p*-value < 0.05. **c**, Retroviral gene transfer of EWS-FLI1 cDNA into MSC lines L87 and V54.2 [4] results in HDAC3 and HDAC8 induction as measured via qRT-PCR, while no change of HDAC1 and HDAC2 expression was observed. Induction of EWS-FLI1-dependent EZH2 expression served as control. **Fig. S2 a**, Tube formation assay with the EwS cell lines CHLA-10 and SK-N-MC after incubation with 3µM MS275 or 4nM FK228 over-night compared to WT control. Both HDACi clearly enhanced endothelial differentiation potential (scale bar 0.5mm). **b**, Analysis of neurogenic differentiation potential of the EwS cell lines CHLA-10, EW7 and SK-N-MC treated for six days with 0.5µM MS-275 or 0.2nM FK228. The neurogenic differentiation marker GFAP (glial fibrillary acidic protein) and GAP43 (growth associated protein 43) were significantly upregulated after incubation with both HDACi as demonstrated by qRT-PCR. **Fig. S3 a**, Cell cycle analysis of CRISPR/Cas9 HDAC1 or HDAC2 knock outs compared to their controls (Cas9) in three different EwS cell lines are shown. Distributional analysis of cell cycle phases of HDAC1 or HDAC2 knock outs compared to their control were performed by propidium iodine staining and flow cytometry measurement, respectively. **b**, To analyze apoptosis in HDAC1 and HDAC2 CRISPR/Cas9 knock outs, DNA double strand breaks were measured with anti-phospho-histone H2AX-FITC conjugated mAbs and counterstained with DAPI. Left, the frequency of g-H2AX positive foci per cell was summarized in bar graphs. Right, fluorescence images show a representative experiment with HDAC1 (top) and HDAC2 (bottom) in two different EwS cell lines each, compared to their controls. **Fig. S4 a**, Western blot analysis of class I HDAC protein levels and their compensation in CRISPR/Cas9 HDAC1 and HDAC2 knock outs compared to their controls (Cas9). Protein levels were detected by antibodies against HDAC1, HDAC2, HDAC3 and HDAC8. b-actin or GAPDH antibodies were used as loading control. **b**, Heat map of 229 genes differentially expressed in three different EwS lines CHLA-10, EW7 and SK-N-MC after CRISPR/Cas9 HDAC1 knock out, are shown. Each column represents one individual array. Microarray data with their normalized fluorescent signal intensities were used (robust multichip average (RMA); GSE162786). **c**, Circos plots of downregulated genes (left column) and heatmaps of pathways and ontology terms the downregulated genes are enriched for (right column). The plots are based on gene lists for three EwS cell lines (CHLA-10, EW7, SK-N-MC), containing the 300 strongest downregulated genes after HDAC1 or HDAC2 knock out, respectively. The lists of downregulated genes for HDAC knock out effects in the top row is based on averaged expression data from HDAC1 and HDAC2 knock outs. The circos plots show overlaps in the gene sets, where each gene is a spot on the inner arc. Purple lines indicate genes shared by the gene lists, and blue lines indicate functional overlaps in the lists. A blue line connects two different genes belonging to the same enriched ontology term. The strongest enriched ontology terms are depicted in the heatmaps. The cells are colored by p-value. Grey cells indicate that a term is not significantly enriched in a gene list. Hence, the heatmap shows common and unique enrichments for the three cell lines. **Fig. S5 a**, HDAC3 or HDAC8 expression after transient shRNA transfection measured by qRT-PCR in EwS cell lines CHLA-10, SK-N-MC or EW7, respectively. Induction of three different shRNAs was done with Doxycycline for 72 hours. **b**, Proliferation of EwS cells after transfection with HDAC3 (top) or HDAC8 (bottom, left) specific shRNA. Further proliferation of SK-N-MC HDAC1 knock out cells with transient HDAC3 or HDAC8 knock down (bottom, right). Control cells were transfected with irrelevant shRNA. Proliferation and cell impendence was measured by the xCELLigence assay every 4 hours. Data are shown as mean ± SEM (hexaplicates/group; *p*-value < 0.001, respectively < 0.0001). **c**, Analysis of the invasive potential of EwS cell line CHLA-10 after transient shRNA transfection with HDAC3 (top) or SK-N-MC HDAC1 knock out with HDAC8 (bottom) specific shRNA 48 hours after seeding. **d**, Evaluation of tumorigenicity of CRISPR/Cas9 knock outs of HDAC1 and their controls (Cas9) in EwS cell line CHLA-10. Immune deficient Rag2-/-γC-/-mice were injected s.c. with 4x10^6^ EwS cells. Mice with an average tumor  size >10 mm in diameter were considered positive and sacrificed. Kaplan-Meier plots of individual experiments with six mice per group are shown. Log-rank test was used to test for differences in survival. **Fig. S6 a**, Proliferation of SK-N-MC and CHLA-10 after treatment with Doxorubicin and/or HDACi (MS-275 or FK228) was analyzed with the xCELLigence system. Cell impedance was measured every 4 hours. Data are shown as mean ± SEM (hexaplicates/group; *p*-value < 0.0001). **Fig. S7** **a**, Proliferation of EwS CRISPR/Cas9 HDAC 1 knock outs and their controls (Cas9) in CHLA-10 or SK-N-MC cells after treatment with Vincristine (top 2 panels) or combined treatment of SK-N-MC with MS-275 and Vincristine (bottom panel). Proliferation and cell impendence were measured by the xCELLigence assay every 4 hours. Data are shown as mean ± SEM (hexaplicates/group; *p*-value > 0.0001). **b**, Heatmaps of pathways and ontology terms that are enriched among up- and downregulated genes. The plots are based on gene lists for two EwS cell lines (EW7, SK-N-MC), containing the 300 strongest differentially expressed genes after FK228, Vincristine or combined treatment, compared to solvent controls, respectively. The strongest enriched ontology terms are depicted in the heatmaps. The cells are colored by p-value. Grey cells indicate that a term is not significantly enriched in a gene list. Hence, the heatmap shows common and unique enrichments for the two cell lines. **c**, Spheroid growth was monitored in Greiner bio-one CELLSTAR® Cell-Repellent Surface 96-well round bottom plates. Left, CHLA-10 or EW7 cells were plated in Matrigel-containing medium and cells were treated for 48 hours with the inhibitors as indicated. Results were compared to solvent controls. Right, primary EwS tumor cells derived from PDX mice. Cell viability was measured with CellTiter Glo® Luminescent assay (quadruplets/group). **Fig. S8 a**, Western blot analysis of apoptosis susceptibility after FK228 or MS-275 and/or A-395 treatment, respectively. Protein levels measured by antibodies against, PARP, CASP3, and GAPDH as loading control. CHLA-10, EW7 or SK-N-MC cells were treated for 48 hours with inhibitors. **b**, Left, heat map of 824 genes, 3-fold differentially expressed in different EwS tumor samples (CHLA-10 and SK-N-MC) at the end of treatment, are shown. Right, zoomed in heat map with 132 genes contains only those genes with a *p*-value < 0.05. Each column represents one individual array. Microarray data with their normalized fluorescent signal intensities were used (robust multichip average (RMA); GSE162788). Cells were treated for 27 hours with solvent control or EEDi (A-395), HDACi (FK228) or with both inhibitors. **c**, Volcano plot of differentially expressed genes of EwS cells at the end of treatment (CHLA-10, SK-N-MC). The plot shows fold changes of log2 expression values (log FC) and p-values obtained from differential expression analysis comparing tumors treated with A-395 + FK228 to solvent controls. Depicted in red are genes obtaining *p*-value < 0.05 and absolute log FC > 1; in blue, genes with *p*-value < 0.05 and absolute log FC ≤ 1; in green, genes with *p*-value ≥ 0.05 and absolute log FC > 1; and in black, genes with *p*-value ≥ 0.05 and absolute log FC ≤ 1. Positive log FCs indicate higher expression of the gene in the treated cell lines. D, GSEA enrichment plots of up- and downregulated gene sets after combined A-395 and FK228 treatment. NES: normalized enrichment score. GSEA: http://www.broadinstitute.org/gsea/index.jsp.

**Additional file 3: Fig. S9. **Whole Western blots with molecular weight markers in selected figures.

## Data Availability

Array data in this manuscript were submitted at Gene Expression Omnibus (GEO) database, https://www.ncbi.nlm.nih.gov/geo/query/acc.cgi?acc=GSE162789. All further data generated or analyzed during this study are included within the article.

## Abbreviations

AKT1: AKT serine/threonine kinase 1
c-myc: MYC protooncogene
Cas9: Type II CRISPR RNA-guided endonuclease
Co-IP: Co-immuno-precipitation
CRISPR/Cas9: Clustered regularly interspaced short palindromic repeats
EED: Embryonic ectoderm development
ETS: Erythroblast transformation specific family
EWSR1: EWS RNA binding protein 1
EZH2: Enhancer of zeste 2 polycomb repressive complex 2 subunit
GAP43: Growth associated protein 43
GFAP: Glial fibrillary acidic protein
GSEA: Gene set enrichment analysis
HDAC: Histone deacetylase
HSP90: Heat shock protein 90
FLI1: Proto-oncogene, ETS transcription factor
p21: Cyclin dependent kinase inhibitor 1A
LSD1: Lysine-specific demethylase 1A
KDM1A: Lysine (K)-specific demethylase 1A
PCR2: Polycomb repressor complex
qRT-PCR: Quantitative real-time PCR
RNAi: RNA interference
SAHA: Suberoylanilide hydroxamic acid
TGF-bRII: Transforming growth factor-beta receptor, type II
TSA: Trichostatin A
