# Correction to: Class I histone deacetylases (HDAC) critically contribute to Ewing sarcoma pathogenesis

**DOI:** 10.1186/s13046-021-02174-4

**Published:** 2022-01-03

**Authors:** Oxana Schmidt, Nadja Nehls, Carolin Prexler, Kristina von Heyking, Tanja Groll, Katharina Pardon, Heathcliff D. Garcia, Tim Hensel, Dennis Gürgen, Anton G. Henssen, Angelika Eggert, Katja Steiger, Stefan Burdach, Günther H. S. Richter

**Affiliations:** 1 Children’s Cancer Research Center and Department of Pediatrics, Klinikum rechts der Isar, Technische Universitat München, München, Germany; 2 German Cancer Research Center (DKFZ), Partner Site Munich, München, Germany; 3 Institute of Pathology, School of Medicine, Technische Universitat München and Comparative Experimental Pathology (CEP), Technische Universitat München, München, Germany; 4 Department of Pediatrics, Division of Oncology and Hematology, Charite - Universitätsmedizin Berlin, Augustenburger Platz 1, Berlin, Germany; 5 Experimental Pharmacology & Oncology Berlin-Buch GmbH, Berlin, Germany

**Correction to: J Exp Clin Cancer Res 40, 322 (2021)**

**https://doi.org/10.1186/s13046-021-02125-z**

Following publication of the original article [1], the authors identified that some of the file descriptions had been omitted from the Additional file 2 and 3 captions.

**Additional file 2: Fig. S1 a**, Expression levels of different class I HDAC genes in different pediatric small-round-blue-cell tumors, carcinomas and normal tissues by box plot presentation using a comparative study of the amc onco-genomics software tool (https://hgserver1.amc.nl/cgi-bin/r2/main.cgi). The number of samples in each cohort is given in brackets. **b**, Differential expression levels of class I HDAC genes in primary EwS at different tumor sites by box plot presentation using the GSE63157 study set and the amc onco-genomics software tool. The number of samples in each cohort is given in brackets, ND: not determined. p-value < 0.05. **c**, Retroviral gene transfer of EWS-FLI1 cDNA into MSC lines L87 and V54.2 [4] results in HDAC3 and HDAC8 induction as measured via qRT-PCR, while no change of HDAC1 and HDAC2 expression was observed. Induction of EWS-FLI1-dependent EZH2 expression served as control.

**Figure S2 a**, Tube formation assay with the EwS cell lines CHLA-10 and SK-N-MC after incubation with 3μM MS275 or 4nM FK228 over-night compared to WT control. Both HDACi clearly enhanced endothelial differentiation potential (scale bar 0.5mm). **b**, Analysis of neurogenic differentiation potential of the EwS cell lines CHLA-10, EW7 and SK-N-MC treated for six days with 0.5μM MS-275 or 0.2nM FK228. The neurogenic differentiation marker GFAP (glial fibrillary acidic protein) and GAP43 (growth associated protein 43) were significantly upregulated after incubation with both HDACi as demonstrated by qRT-PCR.

**Figure S3 a**, Cell cycle analysis of CRISPR/Cas9 HDAC1 or HDAC2 knock outs compared to their controls (Cas9) in three different EwS cell lines are shown. Distributional analysis of cell cycle phases of HDAC1 or HDAC2 knock outs compared to their control were performed by propidium iodine staining and flow cytometry measurement, respectively. **b**, To analyze apoptosis in HDAC1 and HDAC2 CRISPR/Cas9 knock outs, DNA double strand breaks were measured with anti-phospho-histone H2AX-FITC conjugated mAbs and counterstained with DAPI. Left, the frequency of γ-H2AX positive foci per cell was summarized in bar graphs. Right, fluorescence images show a representative experiment with HDAC1 (top) and HDAC2 (bottom) in two different EwS cell lines each, compared to their controls.

**Figure S4 a**, Western blot analysis of class I HDAC protein levels and their compensation in CRISPR/Cas9 HDAC1 and HDAC2 knock outs compared to their controls (Cas9). Protein levels were detected by antibodies against HDAC1, HDAC2, HDAC3 and HDAC8. β-actin or GAPDH antibodies were used as loading control. **b**, Heat map of 229 genes differentially expressed in three different EwS lines CHLA-10, EW7 and SK-N-MC after CRISPR/Cas9 HDAC1 knock out, are shown. Each column represents one individual array. Microarray data with their normalized fluorescent signal intensities were used (robust multichip average (RMA); GSE162786). **c**, Circos plots of downregulated genes (left column) and heatmaps of pathways and ontology terms the downregulated genes are enriched for (right column). The plots are based on gene lists for three EwS cell lines (CHLA-10, EW7, SK-N-MC), containing the 300 strongest downregulated genes after HDAC1 or HDAC2 knock out, respectively. The lists of downregulated genes for HDAC knock out effects in the top row is based on averaged expression data from HDAC1 and HDAC2 knock outs. The circos plots show overlaps in the gene sets, where each gene is a spot on the inner arc. Purple lines indicate genes shared by the gene lists, and blue lines indicate functional overlaps in the lists. A blue line connects two different genes belonging to the same enriched ontology term. The strongest enriched ontology terms are depicted in the heatmaps. The cells are colored by p-value. Grey cells indicate that a term is not significantly enriched in a gene list. Hence, the heatmap shows common and unique enrichments for the three cell lines.

**Figure S5 a**, HDAC3 or HDAC8 expression after transient shRNA transfection measured by qRT-PCR in EwS cell lines CHLA-10, SK-N-MC or EW7, respectively. Induction of three different shRNAs was done with Doxycycline for 72 hours. **b**, Proliferation of EwS cells after transfection with HDAC3 (top) or HDAC8 (bottom, left) specific shRNA. Further proliferation of SK-N-MC HDAC1 knock out cells with transient HDAC3 or HDAC8 knock down (bottom, right). Control cells were transfected with irrelevant shRNA. Proliferation and cell impendence was measured by the xCELLigence assay every 4 hours. Data are shown as mean ± SEM (hexaplicates/group; p-value < 0.001, respectively < 0.0001). **c**, Analysis of the invasive potential of EwS cell line CHLA-10 after transient shRNA transfection with HDAC3 (top) or SK-N-MC HDAC1 knock out with HDAC8 (bottom) specific shRNA 48 hours after seeding. **d**, Evaluation of tumorigenicity of CRISPR/Cas9 knock outs of HDAC1 and their controls (Cas9) in EwS cell line CHLA-10. Immune deficient Rag2-/-γC-/-mice were injected s.c. with 4x10^6^ EwS cells. Mice with an average tumor size >10 mm in diameter were considered positive and sacrificed. Kaplan-Meier plots of individual experiments with six mice per group are shown. Log-rank test was used to test for differences in survival.

**Figure S6 a**, Proliferation of SK-N-MC and CHLA-10 after treatment with Doxorubicin and/or HDACi (MS-275 or FK228) was analyzed with the xCELLigence system. Cell impedance was measured every 4 hours. Data are shown as mean ± SEM (hexaplicates/group; p-value < 0.0001).

**Figure S7 a**, Proliferation of EwS CRISPR/Cas9 HDAC 1 knock outs and their controls (Cas9) in CHLA-10 or SK-N-MC cells after treatment with Vincristine (top 2 panels) or combined treatment of SK-N-MC with MS-275 and Vincristine (bottom panel). Proliferation and cell impendence were measured by the xCELLigence assay every 4 hours. Data are shown as mean ± SEM (hexaplicates/group; p-value > 0.0001). **b**, Heatmaps of pathways and ontology terms that are enriched among up- and downregulated genes. The plots are based on gene lists for two EwS cell lines (EW7, SK-N-MC), containing the 300 strongest differentially expressed genes after FK228, Vincristine or combined treatment, compared to solvent controls, respectively. The strongest enriched ontology terms are depicted in the heatmaps. The cells are colored by p-value. Grey cells indicate that a term is not significantly enriched in a gene list. Hence, the heatmap shows common and unique enrichments for the two cell lines. **c**, Spheroid growth was monitored in Greiner bio-one CELLSTAR® Cell-Repellent Surface 96-well round bottom plates. Left, CHLA-10 or EW7 cells were plated in Matrigel-containing medium and cells were treated for 48 hours with the inhibitors as indicated. Results were compared to solvent controls. Right, primary EwS tumor cells derived from PDX mice. Cell viability was measured with CellTiter Glo® Luminescent assay (quadruplets/group).

**Figure S8 a**, Western blot analysis of apoptosis susceptibility after FK228 or MS-275 and/or A-395 treatment, respectively. Protein levels measured by antibodies against, PARP, CASP3, and GAPDH as loading control. CHLA-10, EW7 or SK-N-MC cells were treated for 48 hours with inhibitors. **b**, Left, heat map of 824 genes, 3-fold differentially expressed in different EwS tumor samples (CHLA-10 and SK-N-MC) at the end of treatment, are shown. Right, zoomed in heat map with 132 genes contains only those genes with a p-value < 0.05. Each column represents one individual array. Microarray data with their normalized fluorescent signal intensities were used (robust multichip average (RMA); GSE162788). Cells were treated for 27 hours with solvent control or EEDi (A-395), HDACi (FK228) or with both inhibitors. **c**, Volcano plot of differentially expressed genes of EwS cells at the end of treatment (CHLA-10, SK-N-MC). The plot shows fold changes of log2 expression values (log FC) and p-values obtained from differential expression analysis comparing tumors treated with A-395 + FK228 to solvent controls. Depicted in red are genes obtaining p-value < 0.05 and absolute log FC > 1; in blue, genes with p-value < 0.05 and absolute log FC ≤ 1; in green, genes with p-value ≥ 0.05 and absolute log FC > 1; and in black, genes with p-value ≥ 0.05 and absolute log FC ≤ 1. Positive log FCs indicate higher expression of the gene in the treated cell lines. **D**, GSEA enrichment plots of up- and downregulated gene sets after combined A-395 and FK228 treatment. NES: normalized enrichment score. GSEA: http://www.broadinstitute.org/gsea/index.jsp

**Additional file 3:**

**Figure S9.** Whole Western blots with molecular weight markers in selected figures.

The supplementary material file captions were updated after initial publication to accurately describe the contents. The correction does not have any effect on the results or conclusions of the paper. The original article has been corrected.
