# Supplementary material for: Class I histone deacetylases (HDAC) critically contribute to Ewing sarcoma pathogenesis
Source: J Exp Clin Cancer Res. 2021 Oct 15;40:322. doi: 10.1186/s13046-021-02125-z (PMC8518288; doi:10.1186/s13046-021-02125-z)
Supplement: Supplementary file 1 — Additional file 1. Supplementary Materials and methods. [file 13046_2021_2125_MOESM1_ESM.docx]

**Additional file 1:**

**Constructs and sgRNAs used**

| **Target gene: HDAC1** |  |
| --- | --- |
| sgRNA target sequence | CATCCGTCCAGATAACATGT |
| sgRNA top (sgHDAC1_6_top) | CACCGCATCCGTCCAGATAACATGT |
| sgRNA bottom (sgHDAC1_6_bottom) | AAACACATGTTATCTGGACGGATGC |
| PAM sequence | CGG |
| Exon number | 3 |
| Rule set 2 score | 0,6964 |

| **Target gene: HDAC2** |  |
| --- | --- |
| sgRNA target sequence | TACAACAGATCGTGTAATGA |
| sgRNA top (sgHDAC2_6_top) | CACCGTACAACAGATCGTGTAATGA |
| sgRNA bottom (sgHDAC2_6_bottom) | AAACTCATTACACGATCTGTTGTAC |
| PAM sequence | CGG |
| Exon number | 6 |
| Rule set 2 score | 0,6176 |

| Gene of interest | Gene set | Vector | Catalog no. | Format |
| --- | --- | --- | --- | --- |
| HDAC8  Homo sapiens  Gene ID: 55869 | shERWOOD UltramiR HDAC8 shRNA Target Gene Set | pZIP-TRE3G-ZsGreen | TLHSU2300-55869-pZIP-TRE3G-ZsGreen-GVO-TRI | Glycerol stock |
| HDAC3  Homo sapiens  Gene ID: 8841 | shERWOOD UltramiR HDAC3 shRNA Target Gene Set | pZIP-TRE3G-ZsGreen | THLSU2300-8841-pZIP-TRE3G-ZsGREEN-GVO-TRI | Glycerol stock |

shRNA sequence CGTCATGTTCTGGTTTGAGAT targeting EWSR1-FLI1 was derived from Broad Institute Clone ID: TRCN0000005322 and used to construct oligos for cloning into pTRIPZmir30 vector [1]. Vector control containing nonsilencing sequence was from Addgene, LGC Standards (Teddington, UK, #127696).

**RNA isolation**

RNA was isolated using TRI Reagent RNA Isolation Kit (Thermo Fisher Scientific) according to the supplier’s recommendations. RNA concentration was measured at 260nm using a nanophotometer (Implen, Munich, Germany) and stored at -80°C and RNA quality was assessed with the Agilent RNA 6000 Nano Kit and the Agilent 2100 Bioanalyzer instrument (Agilent Technologies, California, USA) according to the manufacturer’s instructions.

**Quantitative Real Time-PCR (qRT-PCR)**

Total RNA was reverse transcribed using the High-Capacity cDNA Reverse Transcription Kit (Thermo Fisher Scientific) according to the manufacturer’s instructions. Differential gene expression was then analyzed by qRT-PCR using TaqMan Universal PCR Master Mix and ﬂuorescence detection with a Step One Plus Real-Time PCR (Thermo Fisher Scientific) as described previously [2]. Gene expression was standardized to the housekeeping gene glyceraldehyde-3-phosphate dehydrogenase (GAPDH) and calculated with the 2-ddCt method [3]. All experiments were performed at least in duplicate.

**qRT-PCR assays used**

TaqMan® Gene Expression Assays (Life Technologies) were used for the following genes: HDAC1 (Hs02621185_s1), HDAC2 (Hs00231032_m1), HDAC3 (Hs00187320_m1), HDAC8 (Hs00954353_g1), EZH2 (Hs01016789_m1), GFAP (Hs00909233_m1), GAP43 Hs00176645_m1, and GAPDH (Hs02758991_g1). For the detection of EWS-FLI1 expression, the following primers were designed: 5’-TAGT-TACCCACCCCAAACTGGAT-3’ (sense), 5’-GGGCCGTTGCTCTGTATTCTTAC- 3’ (antisense) and probe 5’-FAM CAGCTACGGGCAGCAGAACCCTTCTT-TAMRA-3’.

**Western blot antibodies**

The following primary antibodies were used: anti-HDAC1 mouse monoclonal antibody (Santa Cruz Biotechnology, Heidelberg, Germany), anti-HDAC2 rabbit monoclonal antibody (Abcam Cambridge, UK), anti-HDAC3 rabbit monoclonal antibody (Abcam), anti-HDAC8 rabbit polyclonal antibody (ThermoFischer Scientific), anti-EED mouse monoclonal antibody (ThermoFischer Scientific), anti-H3K27me3 rabbit polyclonal antibody (Diagenode, Seraing, Belgium), anti-H3K27ac rabbit polyclonal (Abcam), Anti-H3K9/14ac mouse monoclonal antibody (Santa Cruz Biotechnology), anti-PARP rabbit monoclonal antibody (Cell Signaling Technology, Frankfurt am Main, Germany) and anti-Caspase3 rabbit polyclonal antibody (Cell Signaling Technology). Either anti-β-Tubulin rabbit polyclonal antibody (Cell Signaling Technology), anti-Actin mouse monoclonal antibody (Santa Cruz Biotechnology) or anti-GAPDH rabbit monoclonal antibody (Abcam) were used as loading control.

**Differentiation assay**

Cellular tube formation was tested by the use of a commercial Matrigel matrix assay (Biocoat; BD Biosciences) according to the manufacturer’s instruction. Cells were imaged by fluorescence microscopy using a Zeiss AxioVert 100 with AxioVision 4.7.1 software.

**Cell Cycle Analysis**

Knock outs of HDAC1 and HDAC2 in CHLA-10, EW7 and SK-N-MC were harvested and washed three times with cold sample buffer (1g glucose per 1000ml PBS). Supernatant was removed and cells were fixed in 70% ethanol and incubated at 4°C for at least 18 hours. Afterwards cells were centrifuged and stained with PI staining solution (20μg/ml PI; Sigma-Aldrich, St. Louis, MO, USA). RNAse A (ThermoFisher Scientific) 100U/ml sample buffer was added during the staining procedure. Samples and data were analyzed with the FACScalibur^TM^ flow cytometer (BD Biosciences, Franklin Lakes, NJ, USA) using the CellQuest Pro software Version 6.1 (BD Biosciences).

**Gamma H2AX Assay**

HDAC1, HDAC2 CRISPR/Cas9 knock outs or parental Cas9 transfected EwS cells, respectively were seeded in a μ-Slide VI^0.4^ chamber slide (Ibidi, Graefelfing, Germany) allowing the cells to grow adherent for 48 hours. The cells were fixed in 4% paraformaldehyde in PBS for 10 minutes. Afterwards cells were washed with cold PBS and permeabilized with 1% Triton X-100 in PBS at room temperature for 10 minutes. Chambers were incubated with 10% goat serum in PBS for 1 hour. To detect DNA double strand breaks anti-phospho-Histone H2A.X (Ser139) FITC conjugated mouse monoclonal antibody (Merck, Darmstadt, Germany) was used and diluted in 5% goat serum in PBS. After 1 hour, cells were washed 3 times with cold 10% goat serum in PBS for 5 minutes. For staining, one drop of Vectashield Mounting Media with DAPI (Vector laboratories, Burlingame, CA, USA) was used and imaging was done directly by fluorescence microscopy using a Zeiss AxioVert 100 with AxioVision 4.7.1 software.

**Spheroid Growth**

For spheroid formation in Greiner bio-one CELLSTAR® Cell-Repellent Surface 96-well round bottom plates (Kremsmünter, Austria), 100µl of single cell suspension was added per well in medium containing 1x Adv. DMEM/F12 (Life Technologies), B27 (Life Technologies), N-acetyl-L-cysteine (Sigma-Aldrich), A83-01 (Tocris Bioscience), Y-27632 dihydrochloride (Abmole Bioscience) and 2% Matrigel (Corning, NY, USA). The medium was changed every 48-72 hours. Growth was monitored with CellTiter-Glow^R^ 3D (Promega, Madison, WS, USA).

**References:**

1. Chang K, Marran K, Valentine A, Hannon GJ: **Creating an miR30-based shRNA vector**. *Cold Spring Harb Protoc* 2013, **2013**(7):631-635.

2. Richter GH, Fasan A, Hauer K, Grunewald TG, Berns C, Rossler S, Naumann I, Staege MS, Fulda S, Esposito I *et al*: **G-Protein coupled receptor 64 promotes invasiveness and metastasis in Ewing sarcomas through PGF and MMP1**. *The Journal of pathology* 2013, **230**(1):70-81.

3. Livak KJ, Schmittgen TD: **Analysis of relative gene expression data using real-time quantitative PCR and the 2(-Delta Delta C(T)) Method**. *Methods* 2001, **25**(4):402-408.

4. Richter GH, Plehm S, Fasan A, Rossler S, Unland R, Bennani-Baiti IM, Hotfilder M, Lowel D, von Luettichau I, Mossbrugger I *et al*: **EZH2 is a mediator of EWS/FLI1 driven tumor growth and metastasis blocking endothelial and neuro-ectodermal differentiation**. *Proc Natl Acad Sci U S A* 2009, **106**(13):5324-5329.
