# Supplementary figures and images for: Class I histone deacetylases (HDAC) critically contribute to Ewing sarcoma pathogenesis
Source: J Exp Clin Cancer Res. 2021 Oct 15;40:322. doi: 10.1186/s13046-021-02125-z (PMC8518288; doi:10.1186/s13046-021-02125-z)

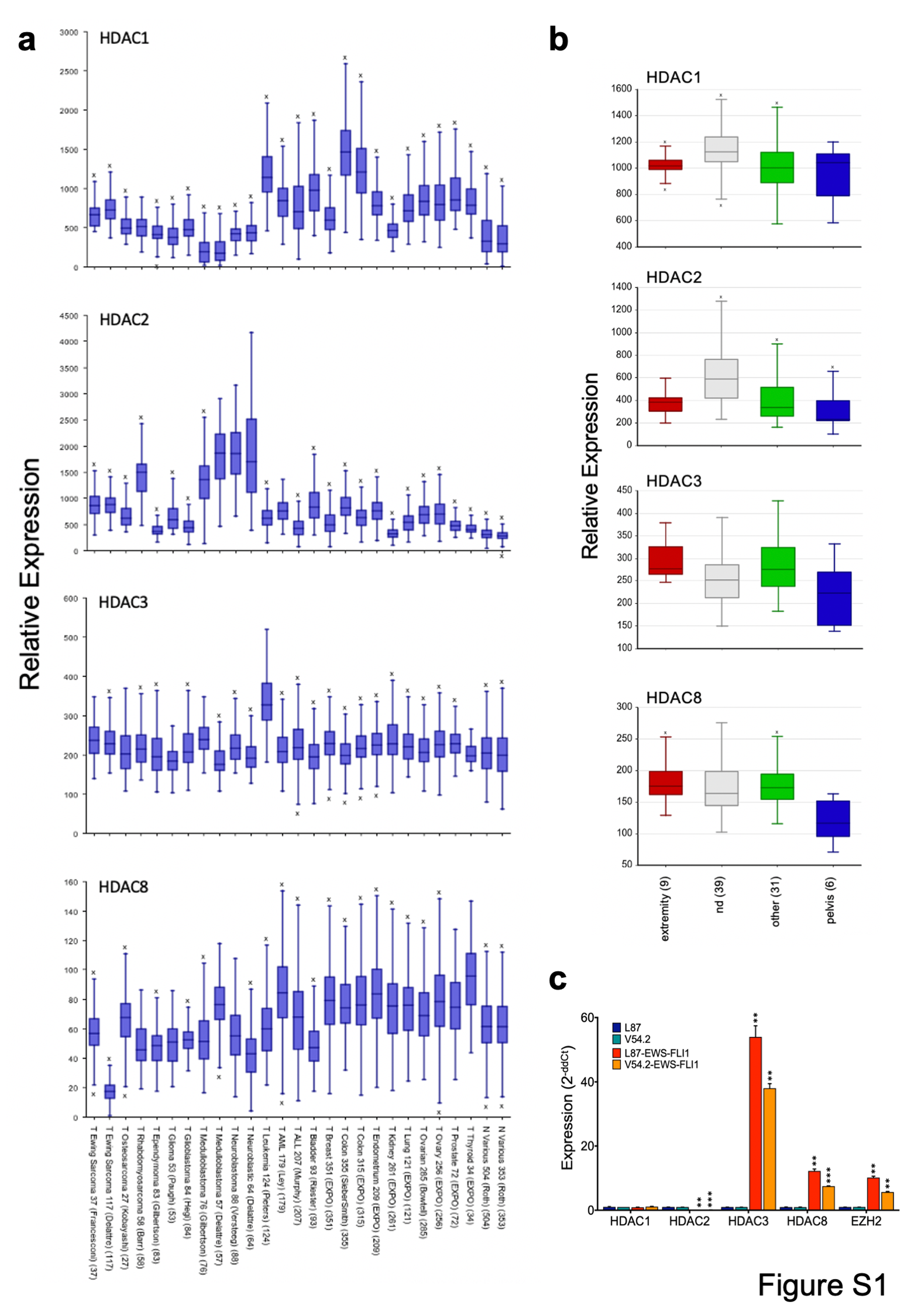

Supplement: Supplementary file 2 — Additional file 2: Fig. S1 a, Expression levels of different class I HDAC genes in different pediatric small-round-blue-cell tumors, carcinomas and normal tissues by box plot presentation using a comparative study of the amc onco-genomics software tool (https://hgserver1.amc.nl/cgi-bin/r2/main.cgi). The number of samples in each cohort is given in brackets. b, Differential expression levels of class I HDAC genes in primary EwS at different tumor sites by box plot presentation using the GSE63157 study set and the amc onco-genomics software tool. The number of samples in each cohort is given in brackets, ND: not determined. p-value < 0.05. c, Retroviral gene transfer of EWS-FLI1 cDNA into MSC lines L87 and V54.2 [4] results in HDAC3 and HDAC8 induction as measured via qRT-PCR, while no change of HDAC1 and HDAC2 expression was observed. Induction of EWS-FLI1-dependent EZH2 expression served as control. Fig. S2 a, Tube formation assay with the EwS cell lines CHLA-10 and SK-N-MC after incubation with 3µM MS275 or 4nM FK228 over-night compared to WT control. Both HDACi clearly enhanced endothelial differentiation potential (scale bar 0.5mm). b, Analysis of neurogenic differentiation potential of the EwS cell lines CHLA-10, EW7 and SK-N-MC treated for six days with 0.5µM MS-275 or 0.2nM FK228. The neurogenic differentiation marker GFAP (glial fibrillary acidic protein) and GAP43 (growth associated protein 43) were significantly upregulated after incubation with both HDACi as demonstrated by qRT-PCR. Fig. S3 a, Cell cycle analysis of CRISPR/Cas9 HDAC1 or HDAC2 knock outs compared to their controls (Cas9) in three different EwS cell lines are shown. Distributional analysis of cell cycle phases of HDAC1 or HDAC2 knock outs compared to their control were performed by propidium iodine staining and flow cytometry measurement, respectively. b, To analyze apoptosis in HDAC1 and HDAC2 CRISPR/Cas9 knock outs, DNA double strand breaks were measured with anti-phospho-histone H2AX-FITC [file 13046_2021_2125_MOESM2_ESM.zip › Figure S1.tif]

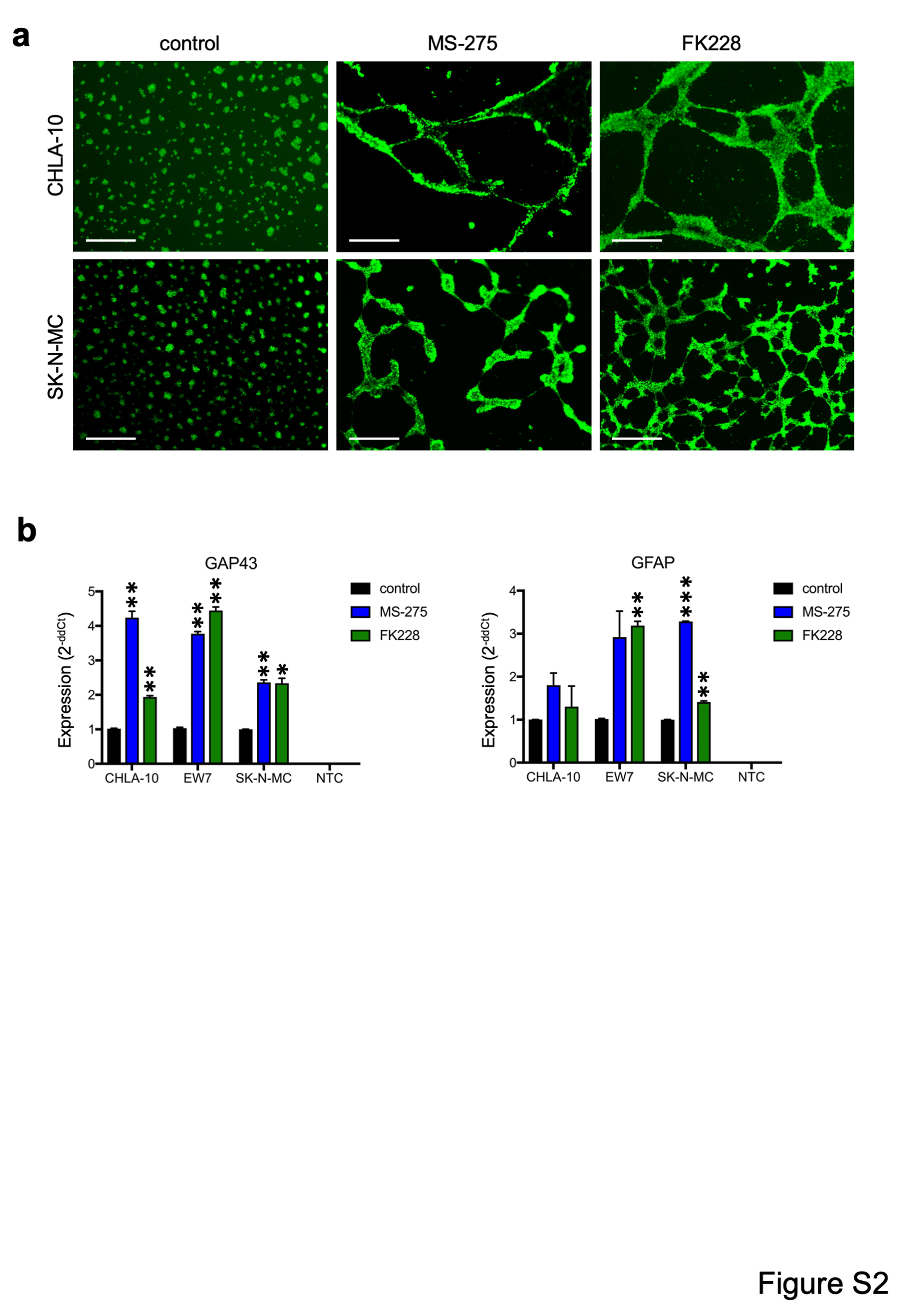

Supplement: Supplementary file 2 — Additional file 2: Fig. S1 a, Expression levels of different class I HDAC genes in different pediatric small-round-blue-cell tumors, carcinomas and normal tissues by box plot presentation using a comparative study of the amc onco-genomics software tool (https://hgserver1.amc.nl/cgi-bin/r2/main.cgi). The number of samples in each cohort is given in brackets. b, Differential expression levels of class I HDAC genes in primary EwS at different tumor sites by box plot presentation using the GSE63157 study set and the amc onco-genomics software tool. The number of samples in each cohort is given in brackets, ND: not determined. p-value < 0.05. c, Retroviral gene transfer of EWS-FLI1 cDNA into MSC lines L87 and V54.2 [4] results in HDAC3 and HDAC8 induction as measured via qRT-PCR, while no change of HDAC1 and HDAC2 expression was observed. Induction of EWS-FLI1-dependent EZH2 expression served as control. Fig. S2 a, Tube formation assay with the EwS cell lines CHLA-10 and SK-N-MC after incubation with 3µM MS275 or 4nM FK228 over-night compared to WT control. Both HDACi clearly enhanced endothelial differentiation potential (scale bar 0.5mm). b, Analysis of neurogenic differentiation potential of the EwS cell lines CHLA-10, EW7 and SK-N-MC treated for six days with 0.5µM MS-275 or 0.2nM FK228. The neurogenic differentiation marker GFAP (glial fibrillary acidic protein) and GAP43 (growth associated protein 43) were significantly upregulated after incubation with both HDACi as demonstrated by qRT-PCR. Fig. S3 a, Cell cycle analysis of CRISPR/Cas9 HDAC1 or HDAC2 knock outs compared to their controls (Cas9) in three different EwS cell lines are shown. Distributional analysis of cell cycle phases of HDAC1 or HDAC2 knock outs compared to their control were performed by propidium iodine staining and flow cytometry measurement, respectively. b, To analyze apoptosis in HDAC1 and HDAC2 CRISPR/Cas9 knock outs, DNA double strand breaks were measured with anti-phospho-histone H2AX-FITC [file 13046_2021_2125_MOESM2_ESM.zip › Figure S2.tif]

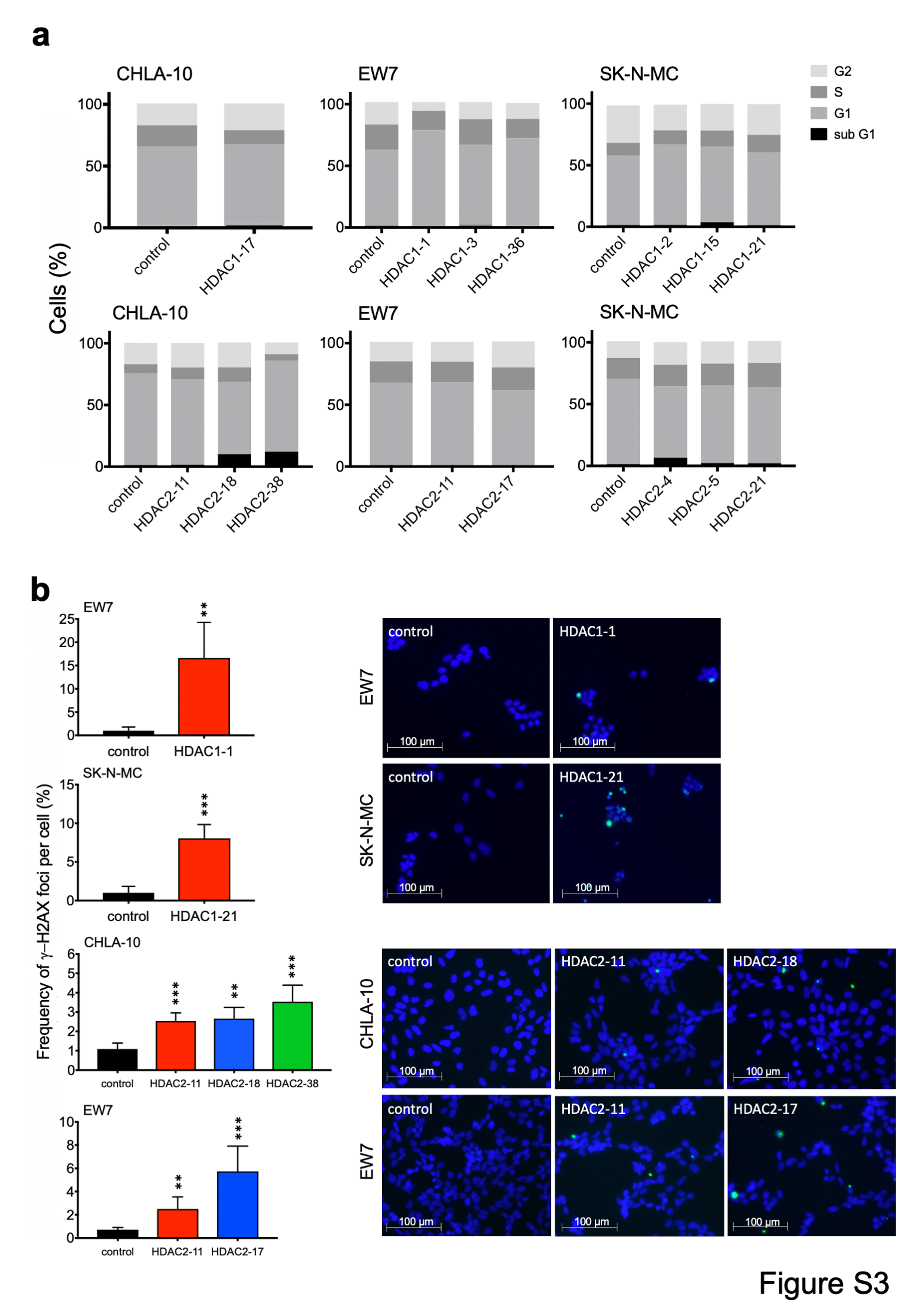

Supplement: Supplementary file 2 — Additional file 2: Fig. S1 a, Expression levels of different class I HDAC genes in different pediatric small-round-blue-cell tumors, carcinomas and normal tissues by box plot presentation using a comparative study of the amc onco-genomics software tool (https://hgserver1.amc.nl/cgi-bin/r2/main.cgi). The number of samples in each cohort is given in brackets. b, Differential expression levels of class I HDAC genes in primary EwS at different tumor sites by box plot presentation using the GSE63157 study set and the amc onco-genomics software tool. The number of samples in each cohort is given in brackets, ND: not determined. p-value < 0.05. c, Retroviral gene transfer of EWS-FLI1 cDNA into MSC lines L87 and V54.2 [4] results in HDAC3 and HDAC8 induction as measured via qRT-PCR, while no change of HDAC1 and HDAC2 expression was observed. Induction of EWS-FLI1-dependent EZH2 expression served as control. Fig. S2 a, Tube formation assay with the EwS cell lines CHLA-10 and SK-N-MC after incubation with 3µM MS275 or 4nM FK228 over-night compared to WT control. Both HDACi clearly enhanced endothelial differentiation potential (scale bar 0.5mm). b, Analysis of neurogenic differentiation potential of the EwS cell lines CHLA-10, EW7 and SK-N-MC treated for six days with 0.5µM MS-275 or 0.2nM FK228. The neurogenic differentiation marker GFAP (glial fibrillary acidic protein) and GAP43 (growth associated protein 43) were significantly upregulated after incubation with both HDACi as demonstrated by qRT-PCR. Fig. S3 a, Cell cycle analysis of CRISPR/Cas9 HDAC1 or HDAC2 knock outs compared to their controls (Cas9) in three different EwS cell lines are shown. Distributional analysis of cell cycle phases of HDAC1 or HDAC2 knock outs compared to their control were performed by propidium iodine staining and flow cytometry measurement, respectively. b, To analyze apoptosis in HDAC1 and HDAC2 CRISPR/Cas9 knock outs, DNA double strand breaks were measured with anti-phospho-histone H2AX-FITC [file 13046_2021_2125_MOESM2_ESM.zip › Figure S3.tif]

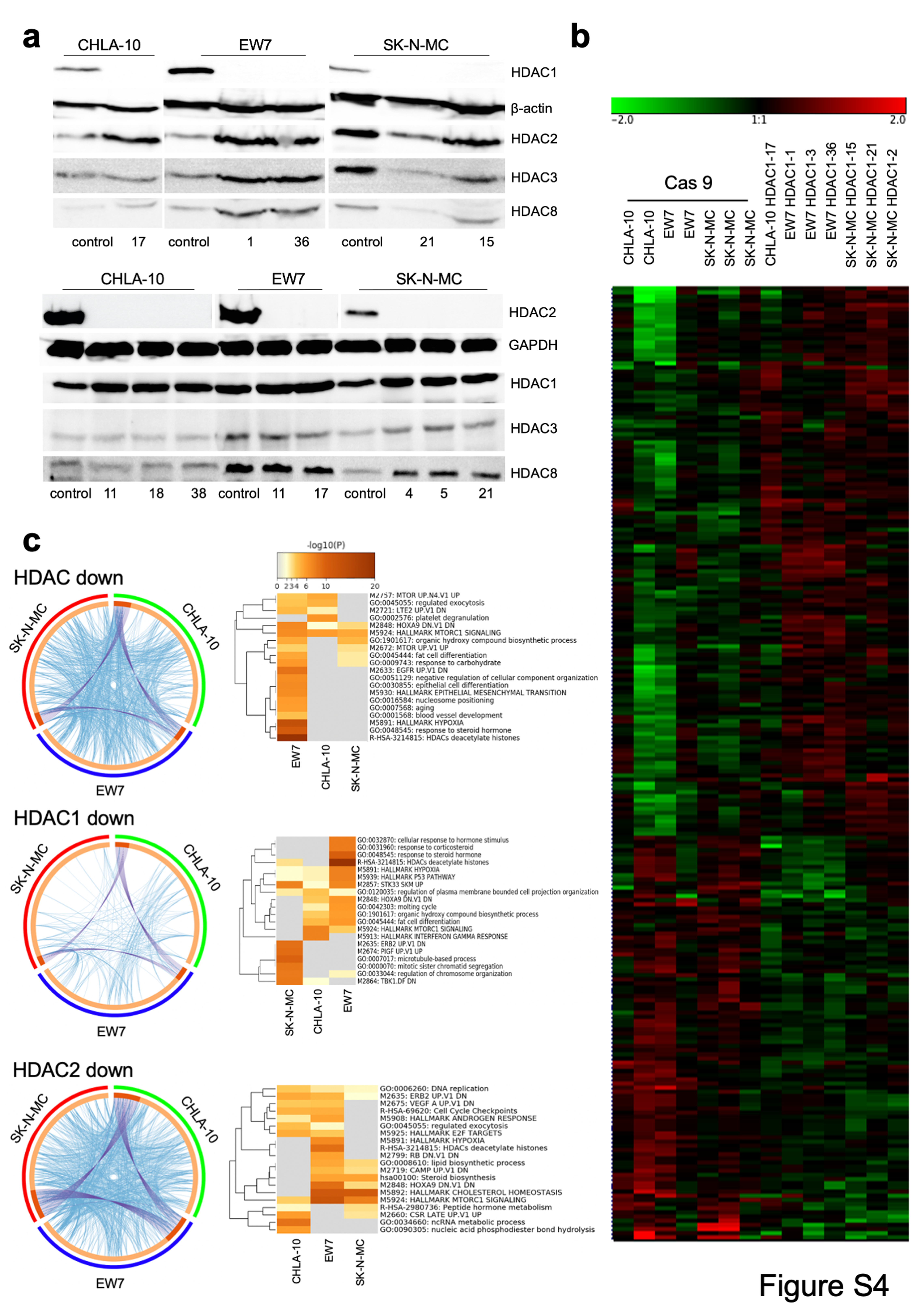

Supplement: Supplementary file 2 — Additional file 2: Fig. S1 a, Expression levels of different class I HDAC genes in different pediatric small-round-blue-cell tumors, carcinomas and normal tissues by box plot presentation using a comparative study of the amc onco-genomics software tool (https://hgserver1.amc.nl/cgi-bin/r2/main.cgi). The number of samples in each cohort is given in brackets. b, Differential expression levels of class I HDAC genes in primary EwS at different tumor sites by box plot presentation using the GSE63157 study set and the amc onco-genomics software tool. The number of samples in each cohort is given in brackets, ND: not determined. p-value < 0.05. c, Retroviral gene transfer of EWS-FLI1 cDNA into MSC lines L87 and V54.2 [4] results in HDAC3 and HDAC8 induction as measured via qRT-PCR, while no change of HDAC1 and HDAC2 expression was observed. Induction of EWS-FLI1-dependent EZH2 expression served as control. Fig. S2 a, Tube formation assay with the EwS cell lines CHLA-10 and SK-N-MC after incubation with 3µM MS275 or 4nM FK228 over-night compared to WT control. Both HDACi clearly enhanced endothelial differentiation potential (scale bar 0.5mm). b, Analysis of neurogenic differentiation potential of the EwS cell lines CHLA-10, EW7 and SK-N-MC treated for six days with 0.5µM MS-275 or 0.2nM FK228. The neurogenic differentiation marker GFAP (glial fibrillary acidic protein) and GAP43 (growth associated protein 43) were significantly upregulated after incubation with both HDACi as demonstrated by qRT-PCR. Fig. S3 a, Cell cycle analysis of CRISPR/Cas9 HDAC1 or HDAC2 knock outs compared to their controls (Cas9) in three different EwS cell lines are shown. Distributional analysis of cell cycle phases of HDAC1 or HDAC2 knock outs compared to their control were performed by propidium iodine staining and flow cytometry measurement, respectively. b, To analyze apoptosis in HDAC1 and HDAC2 CRISPR/Cas9 knock outs, DNA double strand breaks were measured with anti-phospho-histone H2AX-FITC [file 13046_2021_2125_MOESM2_ESM.zip › Figure S4.tif]

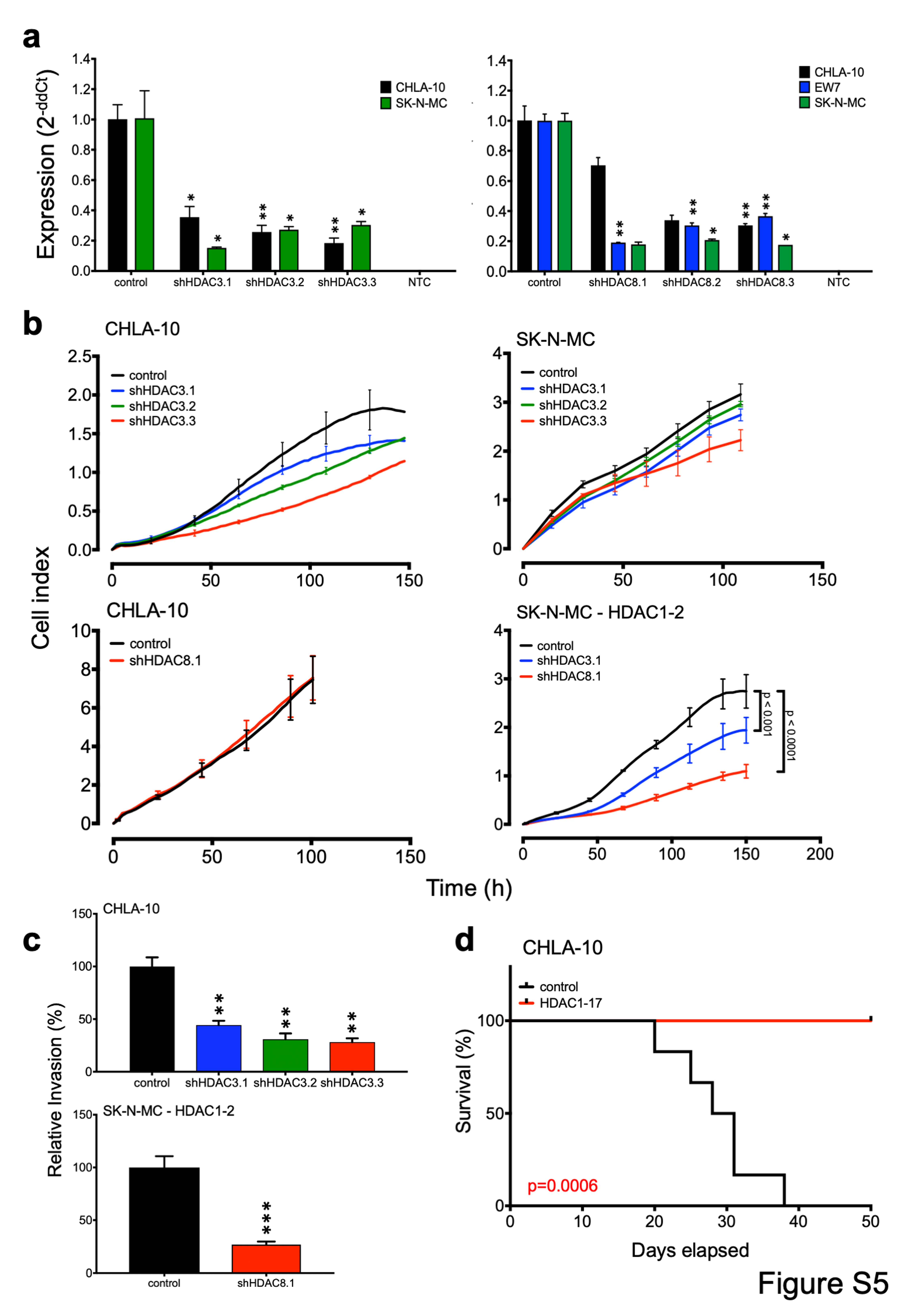

Supplement: Supplementary file 2 — Additional file 2: Fig. S1 a, Expression levels of different class I HDAC genes in different pediatric small-round-blue-cell tumors, carcinomas and normal tissues by box plot presentation using a comparative study of the amc onco-genomics software tool (https://hgserver1.amc.nl/cgi-bin/r2/main.cgi). The number of samples in each cohort is given in brackets. b, Differential expression levels of class I HDAC genes in primary EwS at different tumor sites by box plot presentation using the GSE63157 study set and the amc onco-genomics software tool. The number of samples in each cohort is given in brackets, ND: not determined. p-value < 0.05. c, Retroviral gene transfer of EWS-FLI1 cDNA into MSC lines L87 and V54.2 [4] results in HDAC3 and HDAC8 induction as measured via qRT-PCR, while no change of HDAC1 and HDAC2 expression was observed. Induction of EWS-FLI1-dependent EZH2 expression served as control. Fig. S2 a, Tube formation assay with the EwS cell lines CHLA-10 and SK-N-MC after incubation with 3µM MS275 or 4nM FK228 over-night compared to WT control. Both HDACi clearly enhanced endothelial differentiation potential (scale bar 0.5mm). b, Analysis of neurogenic differentiation potential of the EwS cell lines CHLA-10, EW7 and SK-N-MC treated for six days with 0.5µM MS-275 or 0.2nM FK228. The neurogenic differentiation marker GFAP (glial fibrillary acidic protein) and GAP43 (growth associated protein 43) were significantly upregulated after incubation with both HDACi as demonstrated by qRT-PCR. Fig. S3 a, Cell cycle analysis of CRISPR/Cas9 HDAC1 or HDAC2 knock outs compared to their controls (Cas9) in three different EwS cell lines are shown. Distributional analysis of cell cycle phases of HDAC1 or HDAC2 knock outs compared to their control were performed by propidium iodine staining and flow cytometry measurement, respectively. b, To analyze apoptosis in HDAC1 and HDAC2 CRISPR/Cas9 knock outs, DNA double strand breaks were measured with anti-phospho-histone H2AX-FITC [file 13046_2021_2125_MOESM2_ESM.zip › Figure S5.tif]

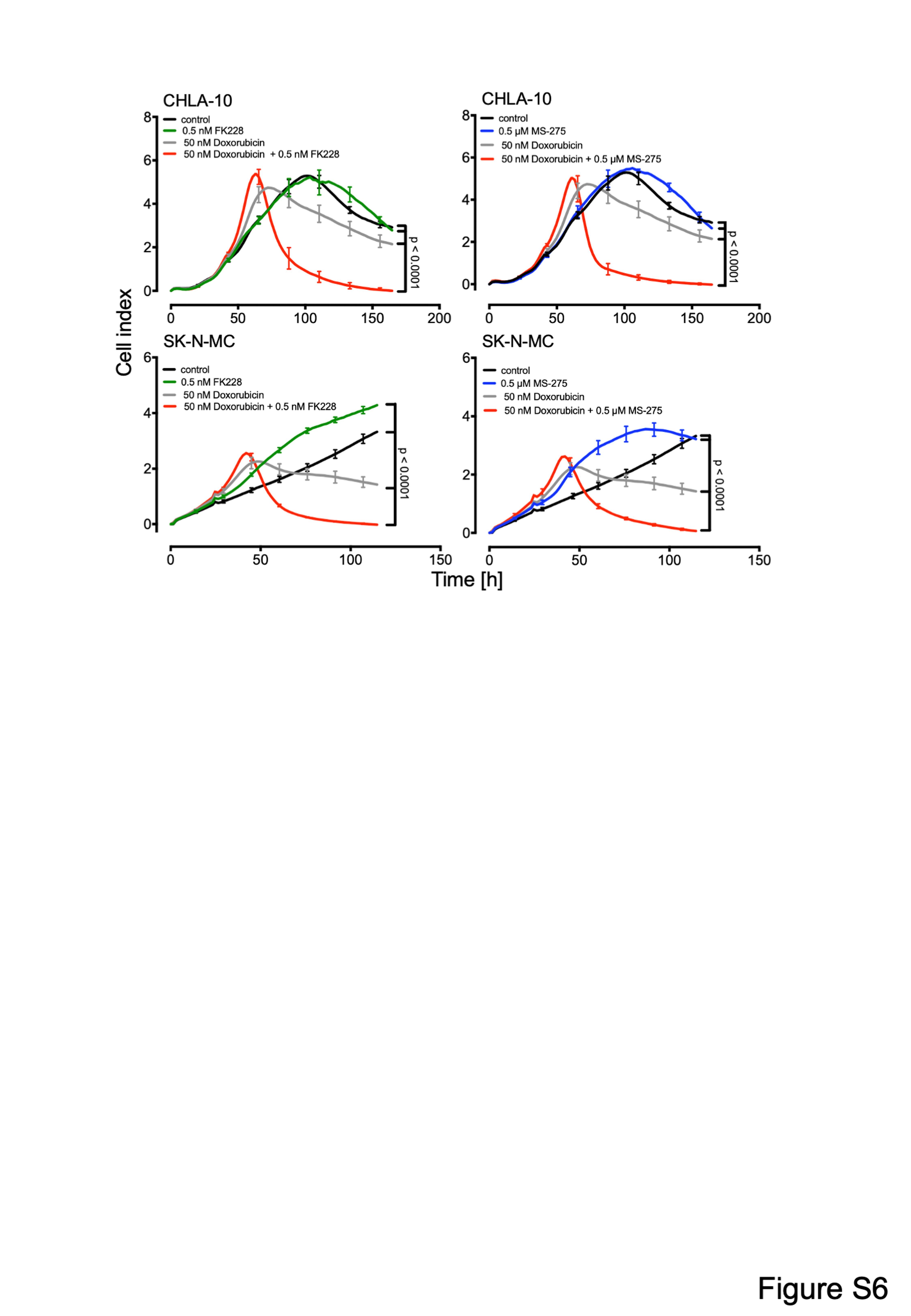

Supplement: Supplementary file 2 — Additional file 2: Fig. S1 a, Expression levels of different class I HDAC genes in different pediatric small-round-blue-cell tumors, carcinomas and normal tissues by box plot presentation using a comparative study of the amc onco-genomics software tool (https://hgserver1.amc.nl/cgi-bin/r2/main.cgi). The number of samples in each cohort is given in brackets. b, Differential expression levels of class I HDAC genes in primary EwS at different tumor sites by box plot presentation using the GSE63157 study set and the amc onco-genomics software tool. The number of samples in each cohort is given in brackets, ND: not determined. p-value < 0.05. c, Retroviral gene transfer of EWS-FLI1 cDNA into MSC lines L87 and V54.2 [4] results in HDAC3 and HDAC8 induction as measured via qRT-PCR, while no change of HDAC1 and HDAC2 expression was observed. Induction of EWS-FLI1-dependent EZH2 expression served as control. Fig. S2 a, Tube formation assay with the EwS cell lines CHLA-10 and SK-N-MC after incubation with 3µM MS275 or 4nM FK228 over-night compared to WT control. Both HDACi clearly enhanced endothelial differentiation potential (scale bar 0.5mm). b, Analysis of neurogenic differentiation potential of the EwS cell lines CHLA-10, EW7 and SK-N-MC treated for six days with 0.5µM MS-275 or 0.2nM FK228. The neurogenic differentiation marker GFAP (glial fibrillary acidic protein) and GAP43 (growth associated protein 43) were significantly upregulated after incubation with both HDACi as demonstrated by qRT-PCR. Fig. S3 a, Cell cycle analysis of CRISPR/Cas9 HDAC1 or HDAC2 knock outs compared to their controls (Cas9) in three different EwS cell lines are shown. Distributional analysis of cell cycle phases of HDAC1 or HDAC2 knock outs compared to their control were performed by propidium iodine staining and flow cytometry measurement, respectively. b, To analyze apoptosis in HDAC1 and HDAC2 CRISPR/Cas9 knock outs, DNA double strand breaks were measured with anti-phospho-histone H2AX-FITC [file 13046_2021_2125_MOESM2_ESM.zip › Figure S6.tif]

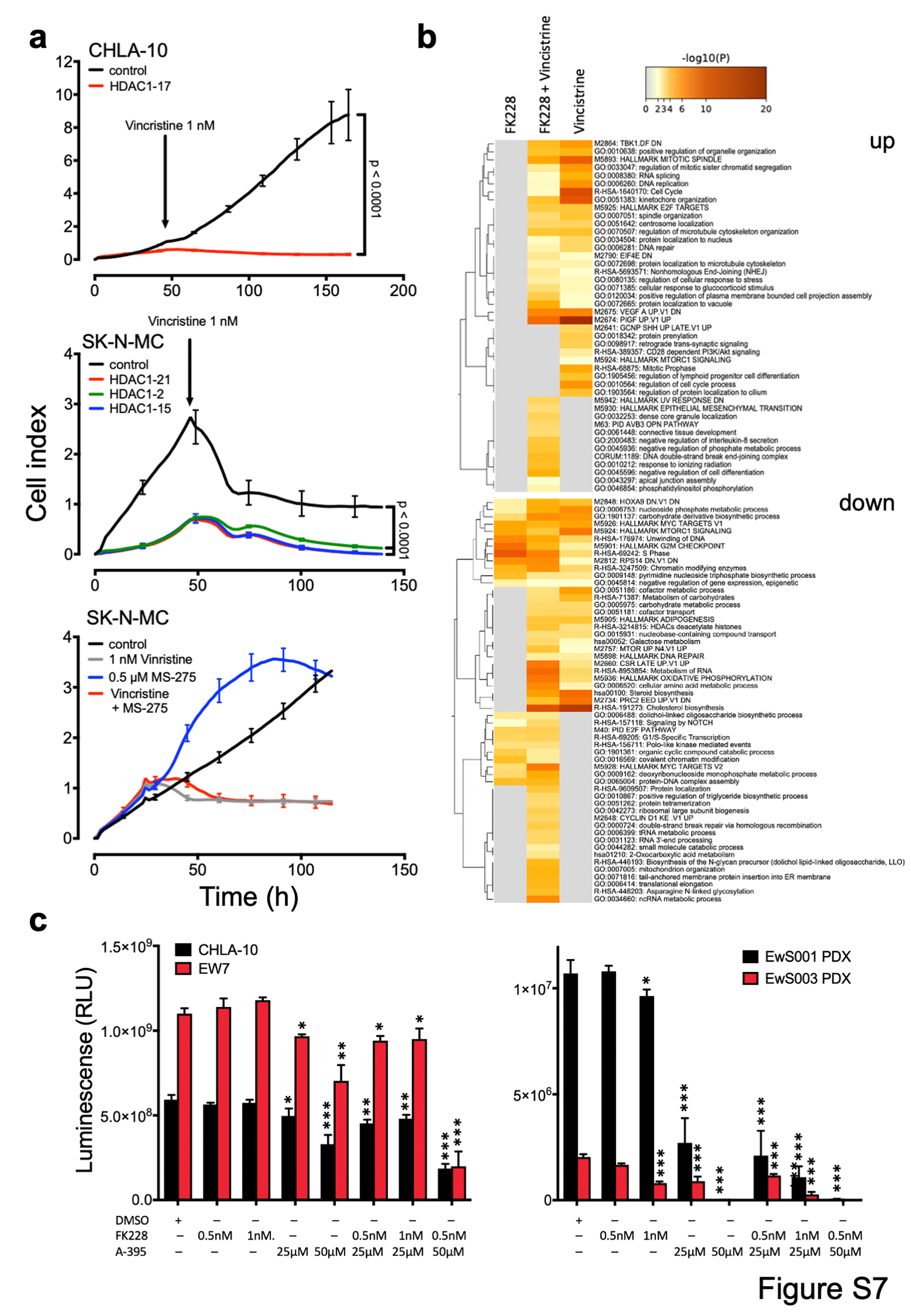

Supplement: Supplementary file 2 — Additional file 2: Fig. S1 a, Expression levels of different class I HDAC genes in different pediatric small-round-blue-cell tumors, carcinomas and normal tissues by box plot presentation using a comparative study of the amc onco-genomics software tool (https://hgserver1.amc.nl/cgi-bin/r2/main.cgi). The number of samples in each cohort is given in brackets. b, Differential expression levels of class I HDAC genes in primary EwS at different tumor sites by box plot presentation using the GSE63157 study set and the amc onco-genomics software tool. The number of samples in each cohort is given in brackets, ND: not determined. p-value < 0.05. c, Retroviral gene transfer of EWS-FLI1 cDNA into MSC lines L87 and V54.2 [4] results in HDAC3 and HDAC8 induction as measured via qRT-PCR, while no change of HDAC1 and HDAC2 expression was observed. Induction of EWS-FLI1-dependent EZH2 expression served as control. Fig. S2 a, Tube formation assay with the EwS cell lines CHLA-10 and SK-N-MC after incubation with 3µM MS275 or 4nM FK228 over-night compared to WT control. Both HDACi clearly enhanced endothelial differentiation potential (scale bar 0.5mm). b, Analysis of neurogenic differentiation potential of the EwS cell lines CHLA-10, EW7 and SK-N-MC treated for six days with 0.5µM MS-275 or 0.2nM FK228. The neurogenic differentiation marker GFAP (glial fibrillary acidic protein) and GAP43 (growth associated protein 43) were significantly upregulated after incubation with both HDACi as demonstrated by qRT-PCR. Fig. S3 a, Cell cycle analysis of CRISPR/Cas9 HDAC1 or HDAC2 knock outs compared to their controls (Cas9) in three different EwS cell lines are shown. Distributional analysis of cell cycle phases of HDAC1 or HDAC2 knock outs compared to their control were performed by propidium iodine staining and flow cytometry measurement, respectively. b, To analyze apoptosis in HDAC1 and HDAC2 CRISPR/Cas9 knock outs, DNA double strand breaks were measured with anti-phospho-histone H2AX-FITC [file 13046_2021_2125_MOESM2_ESM.zip › Figure S7.tif]

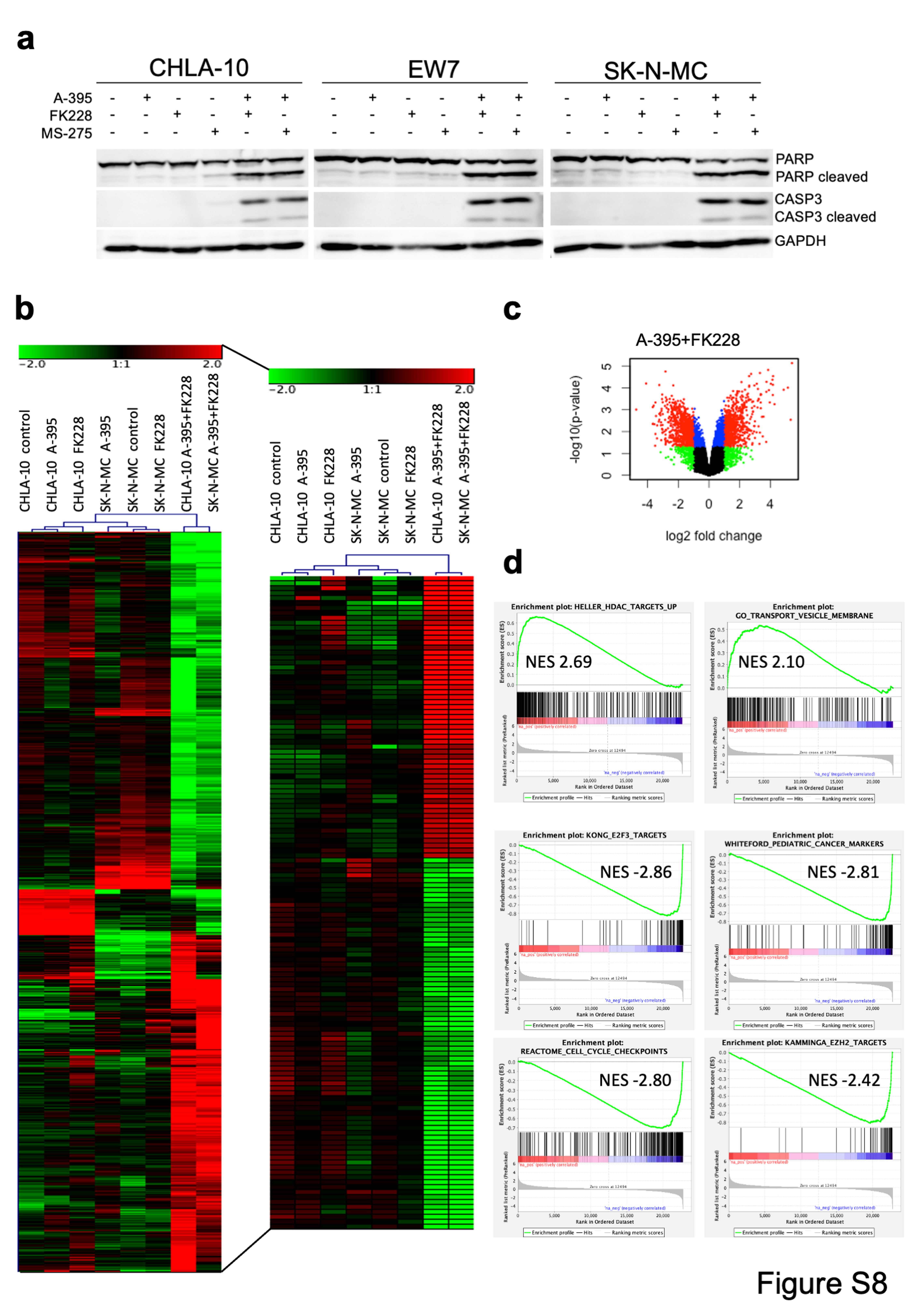

Supplement: Supplementary file 2 — Additional file 2: Fig. S1 a, Expression levels of different class I HDAC genes in different pediatric small-round-blue-cell tumors, carcinomas and normal tissues by box plot presentation using a comparative study of the amc onco-genomics software tool (https://hgserver1.amc.nl/cgi-bin/r2/main.cgi). The number of samples in each cohort is given in brackets. b, Differential expression levels of class I HDAC genes in primary EwS at different tumor sites by box plot presentation using the GSE63157 study set and the amc onco-genomics software tool. The number of samples in each cohort is given in brackets, ND: not determined. p-value < 0.05. c, Retroviral gene transfer of EWS-FLI1 cDNA into MSC lines L87 and V54.2 [4] results in HDAC3 and HDAC8 induction as measured via qRT-PCR, while no change of HDAC1 and HDAC2 expression was observed. Induction of EWS-FLI1-dependent EZH2 expression served as control. Fig. S2 a, Tube formation assay with the EwS cell lines CHLA-10 and SK-N-MC after incubation with 3µM MS275 or 4nM FK228 over-night compared to WT control. Both HDACi clearly enhanced endothelial differentiation potential (scale bar 0.5mm). b, Analysis of neurogenic differentiation potential of the EwS cell lines CHLA-10, EW7 and SK-N-MC treated for six days with 0.5µM MS-275 or 0.2nM FK228. The neurogenic differentiation marker GFAP (glial fibrillary acidic protein) and GAP43 (growth associated protein 43) were significantly upregulated after incubation with both HDACi as demonstrated by qRT-PCR. Fig. S3 a, Cell cycle analysis of CRISPR/Cas9 HDAC1 or HDAC2 knock outs compared to their controls (Cas9) in three different EwS cell lines are shown. Distributional analysis of cell cycle phases of HDAC1 or HDAC2 knock outs compared to their control were performed by propidium iodine staining and flow cytometry measurement, respectively. b, To analyze apoptosis in HDAC1 and HDAC2 CRISPR/Cas9 knock outs, DNA double strand breaks were measured with anti-phospho-histone H2AX-FITC [file 13046_2021_2125_MOESM2_ESM.zip › Figure S8.tif]

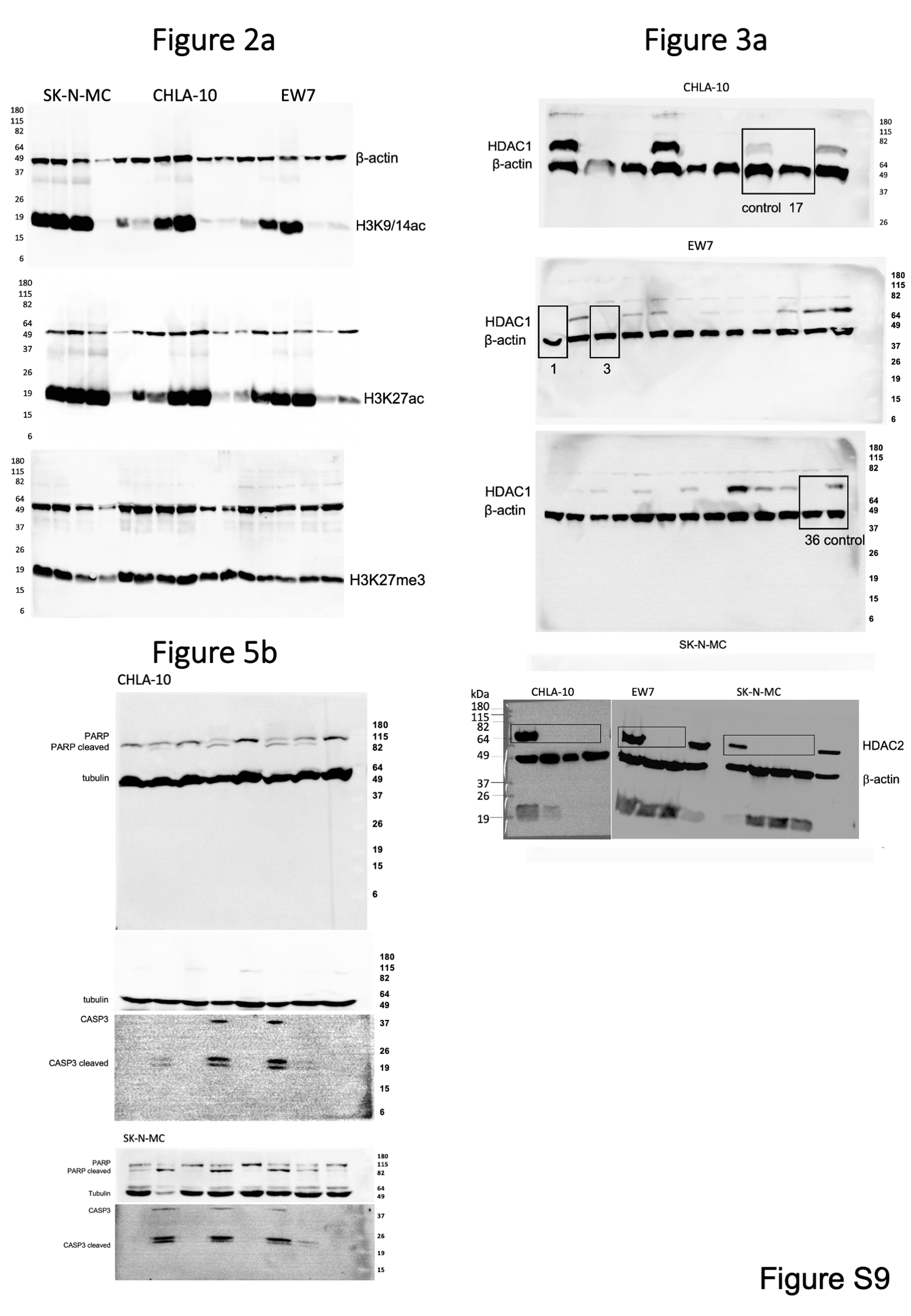

Supplement: Supplementary file 3 — Additional file 3: Fig. S9. Whole Western blots with molecular weight markers in selected figures. [file 13046_2021_2125_MOESM3_ESM.zip › Figure S9-1.tif]

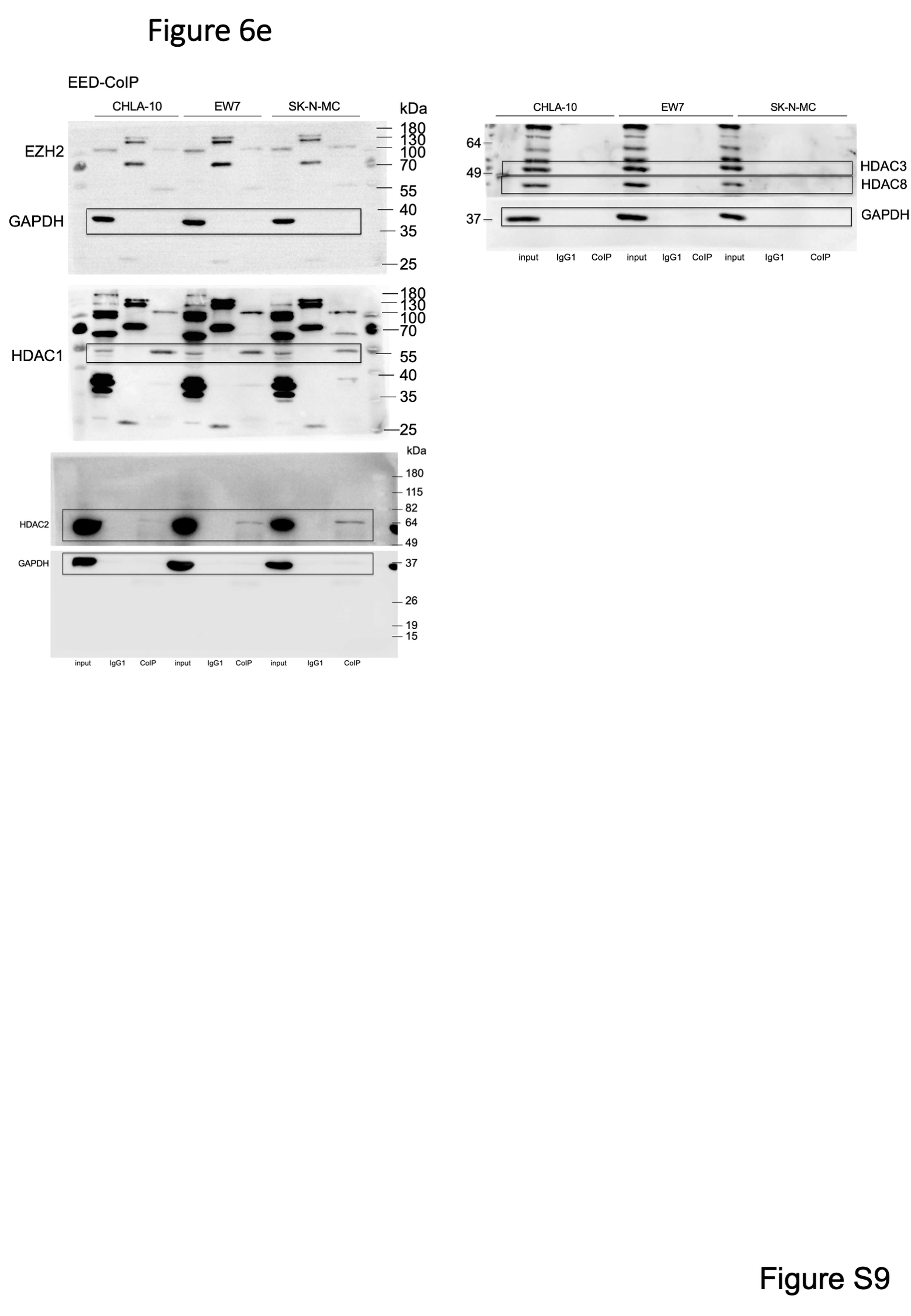

Supplement: Supplementary file 3 — Additional file 3: Fig. S9. Whole Western blots with molecular weight markers in selected figures. [file 13046_2021_2125_MOESM3_ESM.zip › Figure S9-2.tif]

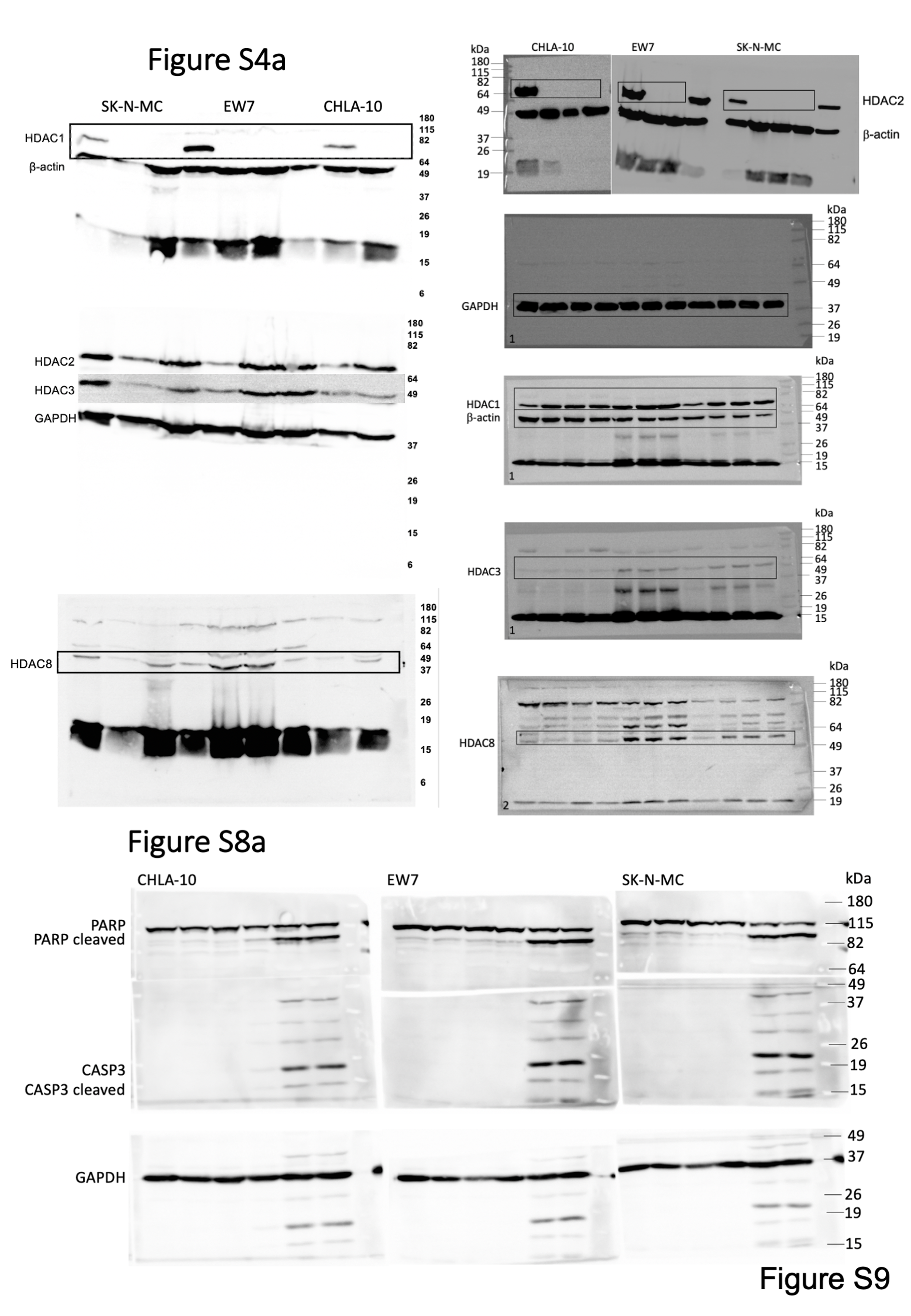

Supplement: Supplementary file 3 — Additional file 3: Fig. S9. Whole Western blots with molecular weight markers in selected figures. [file 13046_2021_2125_MOESM3_ESM.zip › Figure S9-3.tif]
